# Prenatal Cocaine Disrupts Serotonin Signaling-Dependent Behaviors: Implications for Sex Differences, Early Stress and Prenatal SSRI Exposure

**DOI:** 10.2174/157015911796557957

**Published:** 2011-09

**Authors:** Sarah K Williams, Jean M Lauder, Josephine M Johns

**Affiliations:** 1 Curriculum in Neurobiology, University of North Carolina at Chapel Hill, Chapel Hill, NC 27599, USA; 2 Department of Cell and Developmental Biology, University of North Carolina at Chapel Hill, Chapel Hill, NC 27599, USA; 3 Department of Psychiatry, University of North Carolina at Chapel Hill, Chapel Hill, NC 27599, USA; 4 Department of Psychology, University of North Carolina at Chapel Hill, Chapel Hill, NC 27599, USA

**Keywords:** Aggression, depression, development, early environmental stress, prenatal cocaine, prenatal antidepressants, serotonin, sex differences.

## Abstract

Prenatal cocaine (PC) exposure negatively impacts the developing nervous system, including numerous changes in serotonergic signaling. Cocaine, a competitive antagonist of the serotonin transporter, similar to selective serotonin reuptake inhibitors (SSRIs), also blocks dopamine and norepinephrine transporters, leaving the direct mechanism through which cocaine disrupts the developing serotonin system unclear. In order to understand the role of the serotonin transporter in cocaine’s effect on the serotonergic system, we compare reports concerning PC and prenatal antidepressant exposure and conclude that PC exposure affects many facets of serotonergic signaling (serotonin levels, receptors, transporters) and that these effects differ significantly from what is observed following prenatal SSRI exposure. Alterations in serotonergic signaling are dependent on timing of exposure, test regimens, and sex. Following PC exposure, behavioral disturbances are observed in attention, emotional behavior and stress response, aggression, social behavior, communication, and like changes in serotonergic signaling, these effects depend on sex, age and developmental exposure. Vulnerability to the effects of PC exposure can be mediated by several factors, including allelic variance in serotonergic signaling genes, being male (although fewer studies have investigated female offspring), and experiencing the adverse early environments that are commonly coincident with maternal drug use. Early environmental stress results in disruptions in serotonergic signaling analogous to those observed with PC exposure and these may interact to produce greater behavioral effects observed in children of drug-abusing mothers. We conclude that based on past evidence, future studies should put a greater emphasis on including females and monitoring environmental factors when studying the impact of PC exposure.

## A INTRODUCTION

### 1 Prenatal Cocaine Exposure Remains a Public Health Concern

Perception about the impact of prenatal cocaine (PC) exposure on infants and children has evolved since the first studies were performed 30 years ago [1,2]. Initially, there was great concern about “crack babies” and during the mid to late 1980s, the media widely reported major teratological effects, including malformations and withdrawal symptoms [3-6]. Although early case reports showed instances of severe physical teratological abnormalities [7,8], the majority of PC exposed infants exhibited no diagnosable health problems. Developmental studies began to suggest that such severe effects were not as common as once expected, and as the children matured, behavioral effects became less pronounced [9]. However, it is now clear that PC exposure causes reliable and long-lasting behavioral effects, which, although small in magnitude, are still significant [9,10]. In the 1990s, more than 45,000 women reported cocaine use during some period of their pregnancy [11], and gestational cocaine use has continued in the United States, with approximately 5% of pregnant women reporting illicit substance use in 2006 [12]. It has been suggested that PC exposure increases the number of children who need special educational services by as many as 80,550 per year, costing billions in educational and medical services [9,13]. These data highlight the importance of understanding the neurobehavioral outcomes of PC exposure in the hope of developing pharmacological or behavioral therapies for affected children. PC exposure has been extensively studied, providing evidence of disruptions in cardiac, respiratory, renal and neurological functions [14-17], including effects on a number of specific neurobiological systems [18-21]. However, this review will primarily focus on what is known about the effects of PC exposure on the development of the serotonergic system, given that cocaine can act directly on this signaling system. 

### 2 Prenatal Exposure to SSRIs is Widespread Although the Long-term Effects are Unclear

Selective serotonin reuptake inhibitors (SSRIs) like fluoxetine, sertraline and paroxetine act as potent anti-depressants by elevating intrasynaptic levels of serotonin (5- HT) through blocking the ability of the serotonin transporter (SERT) to transport 5-HT into the presynaptic nerve terminal [22]. SSRIs have a greater affinity for SERT, and lower affinities for dopamine (DA) and norepinephrine (NE) transporters compared to cocaine (see Table **1**) [23]. Although a large number of pregnant women use SSRIs to control symptoms of depression and anxiety, little is known about how these drugs may affect the development of the fetal nervous system. Similar to PC exposure, the majority of animal studies have shown no gross teratological effects of prenatal SSRI exposure, although a few studies have reported lower birth weight and/or delayed motor development [24-28]. The safety of human gestational use of these drugs has recently been reviewed and potential risks of major malformations, (e.g., persistent pulmonary hypotension, cardiac defects, miscarriage/spontaneous abortion) from certain SSRIs noted [29-31]. Recently, preclinical studies have begun reporting long-lasting molecular, physiologic and behavioral changes in offspring prenatally exposed to SSRIs [32]. In addition, subtle behavioral changes similar to those observed in PC exposure [9] have been observed in infants prenatally exposed to SSRIs [33], but there have not yet been detailed studies at later ages.

### 3 Changes in Serotonergic Signaling May Underlie Behavioral Changes

**Fig. (1):**
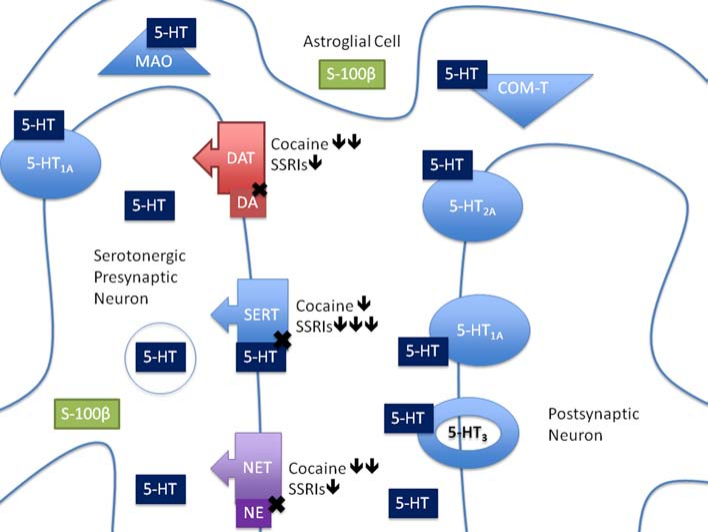
Serotonergic Synapse. This diagram shows a serotonergic presynaptic neuron on the left. This presynpatic neuron is drawn to show release of 5-HT (rectangles) into the synapse. The released 5-HT can act on many substrates. 5-HT can bind to postsynaptic receptors (on right of figure) including g-protein coupled receptors 5-HT_1A_ and 5-HT_2A_ (solid circles) and ionotropic receptors like 5-HT_3_ (hollow circle). 5-HT can act on presynaptic neurons through inhibitory autoreceptors (5-HT_1A_; solid circle in perisynaptic region) and 5-HT transporters. The 5-HT transporter (SERT: arrow box), brings 5-HT back into the axonal bouton. 5-HT is removed from the synapse through metabolic enzymes MAO and COM-T. 5-HT neurons receive trophic signals from astroglial cells during development and possibly throughout life via S100β (rectangle in astroglial cell). This diagram also includes other transporters for comparison. The dopamine transporter (DAT: arrowed box) is responsible for removing dopamine from synapses. The norepinephrine transporter (NET: arrowed box) is responsible for removing norepinephrine from the synapse. All three transporters are blocked by cocaine and to some extent antidepressants. Black arrows represent the relative ability of each compound to affect the transporters (binding affinities can be found in Table 1).

Serotonin is primarily produced by the raphe nuclei in the hindbrain with efferent projections throughout the central nervous system (CNS), mediating and modulating a number of behaviors [34]. The many mechanisms of 5-HT release, auto-regulation, reuptake as well as the many 5-HT receptor subtypes, illustrate the complexity of the serotonergic signaling system, and the diverse ways that cocaine and SSRIs may impact this signaling (Fig. **1**). Nonetheless, it is clear from the available literature that PC exposure has multiple effects on the developing 5-HT system, the extent of which are dependent on sex, age, dose and gestational exposure period, as well as the early postnatal environment. Given the important and dynamic roles of 5-HT in brain development, including autoregulation of its own neuronal development [35], and the complex interactions between receptors and transporters [37-39], it might seem reasonable to attribute differences in serotonergic signaling following PC exposure solely to blockade of SERT. However, the effects of PC exposure on dopamine (DA) and norepinephrine (NE) transporters must also be considered, especially in terms of interference with the actions of stress hormones and promotion of fetal vasoconstriction [39,40]. To address the role of SERT blockade in the serotonergic and behavioral impact of PC exposure, we compare current PC exposure data and effects of prenatal exposure to antidepressants, specifically SSRIs, to provide insights into possible common mechanisms underlying the detrimental effects of PC exposure, and potential therapeutic strategies. 

### 4 Organization of This Review

Throughout this review, we present data from the available literature on the effects of PC exposure followed by the effects of prenatal antidepressant exposure. First, we address effects on the developing serotonergic system. Serotonin is a phylogenetically old signaling molecule with 14 different cognate receptor subtypes, many of which come in a variety of alleles [41-44]. Given the variety of 5-HT receptors, the study of PC exposure has focused mainly on receptors implicated in physiologic or behavioral problems observed in prenatally exposed offspring, namely 5–HT_1A_, 5-HT_2A_, and 5-HT_3_ receptors, which, exhibit very different responses to PC exposure.

 Then we address behavioral alterations following drug exposure and highlight the serotonergic signaling changes that may contribute to these effects. Finally, we present an hypothetical model of how the differences in serotonergic development may contribute to changes in adult serotonergic function and how early environmental stress may interact to compound the negative effects of PC exposure. Additionally, we discuss possible factors influencing vulnerability to the effects of PC exposure including genetic variance and sex. 

This review focuses primarily on the human literature and corresponding preclinical rodent studies. Studies of the effects of PC exposure have also been performed in non-human primates, but have thus far focused on dopaminergic signaling systems. Although several such behavioral studies indicate 5-HT systems are worthy of study few such studies exist to date. All reviewed literature is divided into four postnatal developmental stages: 1) Infancy (defined as postnatal days (PND) 1-10 in the rodent, Age 0-2 years in humans; 2) Juvenile: PND 11-25 in rodent, Age 2-12 in humans; 3) Adolescence: PND 26-50 in rodents, Age 12-20 in humans; 4) Adult: PND 51-forward in rodents, Age 21-forward in humans). Unfortunately, little is known about specific changes in central nervous system (CNS) 5-HT function during the embryonic period when PC or prenatal antidepressant exposure is ongoing. Future studies should address this question to fully determine the time course of effects. 

Table **2** presents a summary of the effects of PC exposure on the 5-HT system components, which is also presented throughout the text. Studies of prenatal drug exposure vary in their dosage and timing of drug administration, and these are important considerations when interpreting effects. 

Experimental design differences are noted in Table **2** and throughout the text for animal literature, since reliable detailed descriptions of dose and length of exposure are not commonly available for clinical datasets. Additionally, the majority of clinical studies combine women on several different types of antidepressants into one group, therefore throughout the review we have listed the drugs included in each study for clarification of potential variability of the effects. It should be noted, however, that a majority of behavioral and biochemical studies have used similar doses, which were initially chosen to mimic doses achieved by drug-abusing women [45].

## B SEROTONERGIC SIGNALING THROUGHOUT DEVELOPMENT

### 1 S100β, a Glial-derived Growth Factor for Serotonin Neurons

Astrocytic release of S100β, regulated by 5-HT_1A_ receptors, acts as a trophic factor for 5-HT neurons, such that less S100β results in reduced survival of these neurons [46]. 5-HT neurons begin differentiation early in gestation and continue through the first postnatal week in rodents [47-49], suggesting that S100β activity remains critical during infancy. S100β can be quantified using techniques such as semi-quantitative immunobinding assays on brain sections [50] or luminescence assays in circulating biological fluids [51]. Additionally, S100β can be localized using immunocytochemical techniques [52].

#### 1.1 Infancy 

##### PC Exposure

Rats prenatally exposed to cocaine (30 mg/kg/day) from gestational day (GD) 1-20 show a significant decrease in S100β immunobinding in the midbrain, and non-significant decreases in the hindbrain (including the raphe nuclei), ventral hippocampus, basal ganglia and forebrain on postnatal days (PND) 1 and 4, and exhibit a trend for decreases on PND 10 in all brain regions assessed [53]. PC exposure for only the last week of gestation (GD 13-20; 40 mg/kg/day), which is equivalent to the second trimester in humans, results in decreases in S100β in the hippocampus and the subplate of cortex in PND 7 rats, which is reversed by 5-HT_1A_ agonists for the first 5 PNDs [52,54]. These PC exposure-dependent decreases in S100β delayed neonatal astroglial development in male rats at PND 6 [55].

##### SSRI Exposure

Chronic prenatal SSRI exposure has been shown to decrease plasma S100β at birth in human infants, suggesting that elevated levels of 5-HT, as a result of blocking reuptake, may down regulate circulating S100β. The impact on CNS S100β and developing 5-HT neurons remains to be determined [56].

#### 1.2 Juvenile, Adolescent and Adult Periods 

##### PC Exposure

The impact of PC exposure on S100β expression in juveniles, adolescents, or adults in either clinical populations or preclinical models has not been assessed. Adult chronic cocaine use does not alter circulating S100β levels in men [51], suggesting that the fetal period may be an especially sensitive window. 

##### SSRI Exposure

Relatively little is known concerning the effects of prenatal SSRI exposure on S100β in juveniles, adolescents or adults. Unlike cocaine, antidepressants can alter circulating S100β when taken during adulthood [57-59], indicating the importance of future studies at these later time points.

#### 1.3 Summary

Given that PC exposure decreases early S100β expression and causes deficits in gliogenesis from infancy that last through adulthood [55,60], and that S100β regulates 5-HT neuronal growth and survival, it is possible that low levels of S100β in infancy may result in long term effects on 5-HT neurons, astrocytes, and associated neuro-glial interactions. Although these effects may be most evident in the raphe nuclei, they likely will also occur in brain regions innervated by 5-HT terminals. Future studies directly measuring the affect of PC exposure on S100β at time points later than infancy in different brain regions will be important to properly evaluate this possibility. 

These data suggest that levels of S100β may be lower during infancy following any type of prenatal SERT blockade (SSRI or cocaine). This could lead to deficits in 5-HT neuronal survival or deficits in associated astrocyte functioning that might persist into adulthood. Future studies should address whether effects observed in infancy are transient or have long-lasting effects. Since S100β serves important functions both centrally and peripherally in the adult nervous system and has been tied to developmental disorders such as schizophrenia, autism, Down Syndrome, depression, and bipolar disorder [61-65], many of which have also been associated with PC exposure (See **Section C. Behavioral Consequences of Prenatal Drug Exposure**), these studies could prove very important in determining the full spectrum of effects of PC and SSRIs on the serotonergic system. 

### 2 Serotonin Levels and Neuronal Growth Throughout Life

Serotonin neurons and fibers can be studied using standard immunocytochemical techniques [54] and quantified using immunobinding methods on tissue sections, whereas 5-HT levels are commonly measured using high-pressure liquid chromatography (HPLC) [66,67]. *p*-Chloroamphetamine (PCA) is a common pharmacologic approach to stimulate release of 5-HT at synapses [68], which has been used effectively to investigate the effects of PC and SSRI exposure on 5-HT release. It is extremely difficult to accurately measure 5-HT (even peripherally) in humans, because of its rapid metabolism. Thus, our knowledge of the effects of PC and SSRIs on human 5-HT content is sparse. However, CNS 5-HT levels can be inferred by monitoring for 5-HT syndrome, a disorder observed in humans and rodents, which exhibit myoclonus, restlessness, tremor, shivering, hyperreflexia, incoordination and rigidity [69]. Therefore, the rodent literature regarding the consequences of PC or prenatal SSRI exposure is primarily reviewed here. 

#### 2.1 Prenatal Period

##### PC Exposure

Following a week of mid-gestational cocaine exposure (GD 8-12; 40 mg/kg/day), 5-HT striatal levels were increased in fetal mice (GD 18), indicating that PC exposure affected late fetal serotonergic development [67]. This is especially noteworthy since SERT expression in 5-HT neurons has not yet begun during this exposure period [70], suggesting that PC exposure alters 5-HT through mechanisms other than CNS SERT blockade during early gestation.

##### SSRI Exposure

SSRIs can block 5-HT uptake into non-neuronal tissues in the craniofacial region or placenta, although their effects on CNS 5-HT is unclear [71-73].

#### 2.2 Infancy 

##### PC Exposure

PC exposure over the last 2 weeks of gestation (GD 7-20; 30 mg/kg/day) does not change spontaneous release of 5-HT, but does result in decreased stimulated 5-HT release in the striatum and nucleus accumbens in both males and females [74], suggesting altered presynaptic regulation. Future studies to determine the effects of PC exposure by direct quantification of 5-HT levels and stimulated 5-HT release in other brain regions, including the raphe nuclei, would help determine whether these effects are specific to the striatum and nucleus accumbens. 

##### SSRI Exposure

No data are currently available regarding changes in 5-HT levels during infancy following prenatal SSRI exposure. However, infants exposed to fluoxetine or citalopram throughout gestation are more likely to exhibit 5-HT syndrome, especially those infants with alleles for slower 5-HT metabolism [75,76].

#### 2.3 Juvenile and Adolescent Periods 

##### PC Exposure

Following PC exposure over 2 weeks in late gestation (GD 13-20; 30 mg/kg/day) or throughout gestation (GD 2-21; 30 mg/kg/day), juvenile male rats showed no differences in 5-HT content in cortex, striatum, hypothalamus or midbrain [66,77]. PCA-stimulated 5-HT release was increased in the midbrain, but not in other brain regions assessed (e.g., cortex, hippocampus, hypothalamus, and striatum) [66]. Although 5-HT levels in the raphe were not assessed, these findings suggest that while production of 5-HT is unaffected by PC exposure, 5-HT release is likely impacted through effects on exocytotic mechanisms, SERT, or inhibitory autoreceptors (5-HT_1A_). Potentially, developmental compensations for increased basal 5-HT levels in infancy could also play a role [74]. PC exposure (GD 8-20; 40 mg/kg/day) was also associated with decreased 5-HT nerve terminals in adolescent male rat hippocampus, with no change detected in cortex [54]. 

##### SSRI Exposure

Following prenatal fluoxetine exposure (GD 13-20; 10 mg/kg/day), no differences were found in 5-HT content (as measured by HPLC) in hippocampus, striatum, or hypothalamus, although decreases in cortical 5-HT were observed in adolescent male rats [78], an effect not observed following PC exposure. Interestingly, prenatal exposure to another antidepressant (amitriptyline; GD 2-21; 10 mg/kg/day), which blocks both 5-HT and NE transporters, decreased basal levels of 5-HT in the striatum (but not prefrontal cortex, hippocampus, hypothalamus, or amygdala) of adolescent rats [78], indicating a complex interaction between serotonergic and noradrenergic mechanisms in the control of 5-HT release.

#### 2.4 Adulthood

##### PC Exposure

Following PC exposure (GD 13-20; 30 mg/kg/day), lower levels of 5-HT were found in the adult rat cerebral cortex and hippocampus, with no effect on the midbrain or hypothalamus, or on PCA-induced 5-HT release in any brain region assessed during adulthood [66]. In contrast, a longer period of PC exposure (GD 2-21; 30 mg/kg/day) in rats is correlated with a transient decrease in striatal 5-HT in early adulthood, that normalized to control levels by late adulthood [77]. These data suggest that 5-HT fibers may be deteriorating with age in the hippocampus and cortex, given that no difference was observed during the juvenile and adolescence time points. 

##### SSRI Exposure

Following prenatal fluoxetine exposure (GD 13-20; 10 mg/kg/day), no differences in 5-HT release were observed in adulthood in any of the same brain regions measured in adolescents, except for a decrease in basal 5-HT in midbrain [78]. Prenatal exposure to amitriptyline (GD 2-21; 10 mg/kg/day) had no effect on 5-HT in male rats in any of the regions assessed during adolescence [77]. There were no changes in PCA-induced 5-HT release in any brain region, except midbrain in adult male rats, where release was decreased by prenatal exposure to fluoxetine, an effect opposite to that seen after PC exposure [78].

#### 2.5 Summary

Studies have thus far focused on male rodents, leaving regional investigation of the effects of PC exposure on 5-HT content and release in females essentially unexplored. This body of evidence indicates that the impact of PC exposure on 5-HT content and release is dependent upon the age of testing and brain region assessed. A more thorough quantification of 5-HT neurons and fibers throughout the brain in both genders is needed, since sex-related behavioral changes are apparent (see** Section C. Behavioral Consequences of Prenatal Cocaine Exposure**). It is clear that effects of PC exposure on basal and stimulated 5-HT release, as well as other signaling systems that impact 5-HT neuronal growth, survival and function may depend on a number of serotonergic factors that can also be disrupted by PC exposure.

Differences between the effects of PC and prenatal SSRI exposure on 5-HT release suggest that PC exposure may impact development of 5-HT neurons through mechanisms other than SERT blockade, especially given the observed impact of very early PC exposure [77]. SERT is not expressed in CNS tissue until GD 12 in rats [70], therefore the action of cocaine on SERT at earlier time points likely occurs through another mechanism, such as cocaine-induced changes in neuronal growth, trophic factors (e.g. S-100β; see **Section B.1.1**)) or 5-HT_1A_ activity (See **Section B.5.1**). 5-HT_1A_ signaling is important for the developmental functions of S100β, which have been shown to be disrupted by PC exposure during infancy [53], a period of rapid neurite outgrowth and synaptogenesis, thus potentially dysregulating appropriate connections or presynaptic mechanisms. Differences observed between cocaine (SERT/NET/DAT blockade), amitriptyline (SERT/NET blockade) and fluoxetine (SERT blockade), highlight the impact of PC exposure on the interaction between multiple monoamine signaling systems during infancy, which may underlie the differences between effects of PC and prenatal SSRI exposure on adult 5-HT levels. For example, NE signaling has been suggested to alleviate some 5-HT syndrome symptoms during infancy [76]. Therefore, a possible explanation for the differences between PC and prenatal SSRI exposure may be the increased synaptic NE. Unfortunately, few studies have investigated this potential mechanism and future work would be greatly informative. 

### 3 Serotonin Metabolism

Serotonin is rapidly metabolized by monoamine oxidase (MAO-A) into a long-lasting compound, 5-Hydroxyindoleacetic acid (5-HIAA), which can be used as a metric for basal 5-HT levels and turnover (5-HT/5-HIAA ratio). In human populations, low plasma and cerebrospinal fluid (CSF) levels of 5-HIAA have been measured with HPLC and tied to suicidal and aggressive behaviors [79,80]. 

#### 3.1 Infancy

##### PC Exposure

Human PC exposed infants show no differences in CSF levels of 5-HIAA [81], and no published results exist that utilize animal models to study infant levels of 5-HIAA. However, given the suggestion that 5-HT levels are lower during infancy following PC exposure, it is reasonable to hypothesize that 5-HIAA would be lower as well, although this has not been directly tested. Additionally, cocaine can increase MAO-A activity, which is interesting since MAO-A is expressed as early as GD 12 in rats [67,82,83], providing a possible mechanism through which PC exposure could impact 5-HT metabolism. Fetal 5-HIAA measurements could be obtained given the fact that increased 5-HT has been observed during the fetal period following PC exposure in mice [67]. Taken together, these data would suggest a possible disruption in fetal 5-HT metabolism by PC exposure.

##### SSRI Exposure

No studies have been published regarding infant levels of 5-HIAA in animal models following prenatal SSRI exposure. Infants exposed to either fluoxetine or citalopram throughout gestation show reduced 5-HIAA in the cord blood in the first few days of life [75].

#### 3.2 Juvenile and Adolescent Periods

##### PC Exposure

In juvenile male and female rats, PC exposure (GD 2-21;10 mg/kg/day) or (GD 13-20; 30 mg/kg/day) does not significantly alter 5-HIAA levels in the striatum, hippocampus, prefrontal cortex or midbrain [66,77]. These data are similar to those found for the effect of PC exposure on 5-HT, suggesting that 5-HT metabolism is likely unaffected at this age.

##### SSRI Exposure

Prenatal fluoxetine exposure (GD 13-20; 10 mg/kg/day) does not affect 5-HIAA or the 5-HT/5-HIAA ratio in the cortex, hippocampus, striatum, midbrain or hypothalamus during adolescence in male rat offspring [78]. 

#### 3.3 Adulthood

##### PC Exposure

Male and female rats exhibit no differences in 5-HIAA levels, other than a reported decrease in male hypothalamus by adulthood following PC exposure (GD 2-21; 30 mg/kg/day) [77]. This is particularly intriguing given that with the same PC exposure, adult rats had lower 5-HT levels in the hippocampus and cortex (see **Section B. 2.4 in Serotonin Levels and Neuronal Growth Throughout Life**). This suggests that there may be changes in the rate of 5-HT metabolism in PC exposed rodents that does not appear until adulthood and that is regionally specific.

##### SSRI Exposure

Prenatal fluoxetine exposure (GD 13-20; 10 mg/kg/day) in rats had no effect on 5-HIAA levels in any brain region [78]. Since there is decreased 5-HT in the midbrain of adult rats, this indicates a potential deficit in metabolism due to prenatal SSRI exposure that may be specific to the midbrain [78]. Exposure to amitriptyline (GD 2-21; 10 mg/kg/day) during the same period results in a transient decrease in striatal 5-HIAA at PND 60 in male rats, similar to what is observed with 5-HT levels, suggesting no effect on 5-HT metabolism [77].

#### 3.4 Summary

PC exposure seems to impact the hypothalamus and hippocampus to a greater extent than other brain regions [77]. This suggests that interactions between cellular signaling components may differ in these regions in response to SERT blockade, although this needs to be directly tested. Future studies could also explore whether exposure to SSRIs would cause similar changes in males and females following longer exposure paradigms. 

Interestingly, susceptibility to the negative impact of prenatal SSRI exposure in human infants appears to be dependent on genetic variance in MAO-A and c-O-methyltransferase (COMT), another 5-HT metabolic enzyme. Specifically, individuals who rapidly metabolize 5-HT when exposed to SSRIs show greater 5-HT syndrome symptoms [76]. Given that cocaine can enhance MAO-A activity [82], both PC and prenatal SSRI exposure appear to result in regionally-specific changes in 5-HT metabolism (see **Section B.2.4 Serotonin Levels and Neuronal Growth Throughout Life**), and changes in 5-HT metabolism that are involved in depression and aggression [79,80]. Children with these exposure histories should be carefully monitored as they mature. 

### 4 Developmental Effects on Serotonin Reuptake Sites

Sites of 5-HT reuptake (expression of the serotonin transporter, SERT) are important for maintaining serotonergic tone by recycling synaptic 5-HT back into presynaptic serotonergic nerve terminals. Importantly, these are sites of direct action of both SSRIs, and non-selective serotonin reuptake inhibitors (SRIs) including cocaine, which bind and block 5-HT transport [39,84]. SERT can be measured by immunobinding or binding assays using known SERT binding drugs such as, citalopram or paroxetine, and is found throughout the brain and is well known for its involvement in mood regulation [54,78,84]. Not surprisingly, SERT is impacted by prenatal exposure to both cocaine and SSRIs.

#### 4.1 Infancy 

##### PC Exposure

PC exposure (GD 13-20; 30 mg/kg/day) in rats causes a decrease in cortical and hippocampal 5-HT uptake sites from PND 1-7 in both males and females [54,85]. Moreover, reduced citalopram binding is seen in the striatum and nucleus accumbens, but not the midbrain, of males and females at PND 7 following a longer PC exposure (GD 7-20; 30 mg/kg/day ) [74]. 

##### SSRI Exposure

To date there are no studies that have directly investigated the effects of SSRIs on SERT expression or activity during infancy in either clinical or preclinical models.

#### 4.2 Juvenile and Adolescent Periods 

##### PC Exposure

PC exposure (GD 8-20; 40 mg/kg/day) results in increased SERT binding in the nucleus accumbens without affecting the striatum or hippocampus during the juvenile period [86]. Studies measuring increased PCA-stimulated 5-HT release suggested that PC exposure (GD 13-20; 30 mg/kg/day) may also increase SERT expression in the midbrain of juvenile male [66]. This reliable method of increasing synaptic 5-HT is partially driven by PCA competitively antagonizing SERT; therefore, a greater number of PCA action sites indirectly suggests more SERT to explain the enhanced 5-HT release [68]. In contrast, during adolescence, PC exposure (GD 13-20; 40 mg/kg/day) decreased SERT binding in the nucleus accumbens in male rats, with no effect in the hippocampus or striatum [54]. 

##### SSRI Exposure

Fluoxetine (GD 1-21; 2.5 mg/kg/day) and chlorimpramine (GD 1-21; 10 mg/kg/day) result in 30% reduction in cortical SERT binding sites at PND 25 [87]. Although early studies found no differences in SERT in the cortex, striatum, hippocampus, midbrain or hypothalamus of juvenile male rats following prenatal fluoxetine exposure (GD 13-20; 10 mg/kg/day) [78], more detailed anatomical analyses have indicated increased expression during the juvenile period in the basolateral and medial amygdala, CA2 and CA3 regions of the hippocampus and the lateral hypothalamus. In contrast, a decrease in SERT expression was reported in the dorsomedial hypothalamus in the same animals [88]. These data suggest that the length of exposure or dose may play important roles in the development of SERT expression, especially in the cortex since opposite results were reported in these studies. More anatomically detailed investigations following various exposure lengths and doses could determine the full impact of SSRI exposure on juvenile SERT.

#### 4.3 Adulthood 

##### PC Exposure

PC exposure (GD 8-20; 40 mg/kg/day) has no impact on SERT expression in the striatum, nucleus accumbens or hippocampus of adult rats [86]. Surprisingly, little is known about the impact of PC exposure on adult SERT expression in other brain regions, particularly cortex where effects are seen earlier in development. Moreover, the studies performed thus far have not included females, although SERT activity certainly varies across gender [89,90].

##### SSRI Exposure

Several studies have reported no change in SERT expression in adult rats following prenatal fluoxetine exposure (GD 13-20; 10 mg/kg/day) [78,88]. Early studies measured SERT in the cortex, striatum, hippocampus, midbrain, and hypothalamus [78], and later reports focused on specific sub-regions including: the dorsal and medial raphe nuclei; lateral and dorsomedial nuclei of the hypothalamus; the CA3 and CA2 regions of the hippocampus, and the basolateral and medial nuclei of the amygdala [88]. Fluoxetine (GD 1-21; 2.5 mg/kg/day) and chlorimpramine (GD 1-21; 10 mg/kg/day) do not effect cortical SERT binding sites at PND 90 [87]. These negative results mirror those observed following PC exposure. However, a recent study has shown a 35% decrease in SERT immunoreactivity in the raphe nuclei of rats on PND 120 following fluoxetine exposure from GD 14-PND 7 [91].

#### 4.4. Summary

Taken together, these results suggest that PC exposure studies should investigate changes in SERT expression with higher anatomical resolution in the future, similar to studies with prenatal SSRIs, where differences emerged only when brain regions were examined in more detail. Adolescence might prove to be a particularly good developmental time point for such analysis, since the greatest differences were seen following SSRI exposure in this group. Additionally, it would appear that early disruption (until early adolescence) of SERT expression normalizes by adulthood, with the nucleus accumbens exhibiting the greatest change throughout development. Studies that pinpoint developmentally dynamic changes in SERT expression and activity following PC exposure would be informative, especially in the context of SERT-dependent behavioral changes. Few studies have examined the impact of prenatal exposure to either cocaine or SSRIs on female SERT expression. Given that SERT is regulated differently in males and females [38,89,92,93], it will be important to determine if females are equally impacted by PC or prenatal SSRIs.

### 5 5-HT_1A_ Receptors

5-HT_1A_ receptors serve a variety of functions, acting as both presynaptic autoreceptors on 5-HT neurons in the raphe nuclei [94] and as postsynaptic receptors on neurons innervated by 5-HT axons throughout the brain, particularly in limbic areas, cingulate and prefrontal cortices [34,95]. These receptors are also expressed on astrocytes, which release S-100β, a growth factor for 5-HT neurons, as discussed above. Additionally, 5-HT_1A_ receptors in the hypothalamus contribute to stress-induced release of adrenocorticotrophic hormone (ACTH), corticosterone (the rodent equivalent of cortisol), renin, prolactin and oxytocin into the bloodstream by the pituitary [96]. Consequently, this receptor has received the most attention in PC exposure studies. 

#### 5.1 Infancy 

##### PC Exposure

**Fig. (2):**
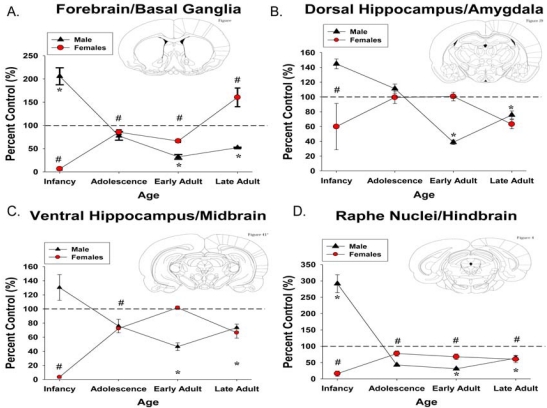
The Impact of Prenatal Cocaine Exposure on 5-HT_1A_ Receptors is Region-Specific. Immunobinding data are represented as percent control means ± SEM (i.e. PC exposed males compared to Control Males). Asterisks (*) represent that PC exposed males differed significantly from control males (p ≤ 0.05). Pound signs (#) represent that PC exposed females differed significantly from control females (p ≤ 0.05). Infancy: PND 1, Adolescence: PND 30, Early Adult: PND 60, Late Adult: PND 120. Insets display representative anatomical sections from which immunobinding data was collected. PND: postnatal day.

Following full gestational PC exposure (GD 1-20; 30 mg/kg/day), male rats on PND 1 show significantly increased 5-HT_1A_ receptors in the raphe nuclei, basal ganglia and forebrain, and non-significant increases in the hippocampus and substantia nigra as measured by semi-quantitative^ 125^I-protein A immunobinding (Fig. **2**), in conjunction with an increase in 5-HT_1A_ mRNA in brainstem [50,53]. However by PND 10, 5-HT_1A_ immunobinding normalizes in the midbrain, but continues to show decreases in the basal ganglia/forebrain and hindbrain in conjunction with reduced 5-HT_1A_ mRNA expression [53]. In contrast, females exhibit decreased 5-HT_1A_ receptors in the raphe nuclei, hippocampus, basal ganglia and forebrain on PNDs 1 and 10 [50,53] (see Fig. **2**). 

##### SSRI Exposure

To date there are no studies that have directly investigated the effects of SSRIs on 5-HT_1A_ receptors expression or activity during infancy in either clinical or preclinical models. 

#### 5.2 Juvenile and Adolescent Periods 

##### PC Exposure

PC exposure (GD 13-20; 30 mg/kg/day) does not affect 5-HT_1A_ receptors in cortex, hypothalamus or midbrain of prepubescent male or female rats, as measured by radioligand binding assays [85,97]. This exposure paradigm does result in increased 5-HT_1A_-stimulated release of ACTH and renin in juvenile males, but not females [85], without impacting basal release. These data suggest altered activity without a concurrent change in expression. Following longer PC exposure (GD 1-20; 30 mg/kg/day) male 5-HT_1A_ immunobinding throughout the brain is not altered during adolescence (PND 30), although females show reduced receptor immunobinding across all brain regions examined with the exception of the dorsal hippocampus and amygdala [53,50] (see Fig. **2**). 

##### SSRI Exposure

To date there are no studies that have directly investigated the effects of SSRIs on 5-HT_1A_ receptor expression or activity during juvenile or adolescent periods in either clinical or preclinical models.

#### 5.3 Adulthood 

##### PC Exposure

**Fig. (3):**
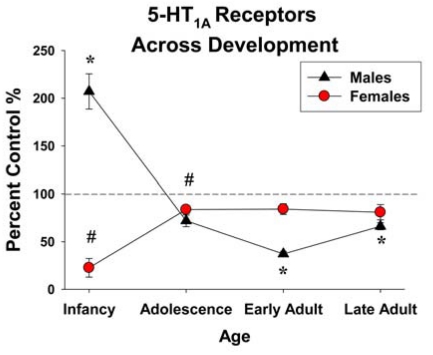
Whole brain 5-HT_1A_ receptor immunobinding. Represented as percent control means ± SEM (i.e. PC exposed males compared to control males) for each sex. Asterisks (*) represent that PC exposed males differed significantly from control males (p ≤ 0.05). Pound signs (#) represent that PC exposed females differed significantly from control females (p ≤ 0.05). Infancy: PND1, Adolescence: PND 30, Early Adult: PND 60, Late Adult: PND 120 PND: postnatal day.

PC exposure (GD 13-20; 30 mg/kg/day) does not affect 5-HT_1A_ receptors in the cortex, hypothalamus or midbrain of adult male or female rats, as measured by radioligand binding assays at PND 70 [85,97]. Receptor dynamics (production, degradation and half life) are also unaffected in adulthood by this exposure paradigm [98]. Unlike adolescents, adults show no change in 5-HT_1A_-stimulated release of any hypothalamic hormone, and also have normal basal hormone levels [85]. Chronic paroxetine administration desensitizes 5-HT_1A _receptors [99] and PC exposure (GD 13-20; 30 mg/kg/day) decreases 5-HT_1A_-stimulated ACTH release following a 2 week paroxetine treatment period in adult male but not female rats, indicating a differential, sex-specific sensitivity to alterations in serotonergic signaling following PC exposure [100]. PC exposure (GD 1-20; 30 mg/kg/day) alters brain 5-HT_1A_ expression in a sex-specific manner (Figs. **2** and **3**), as measured by semi-quantitative^ 125^I-protein A immunobinding [50,53]. During early adulthood (PND 60), males have decreased 5-HT_1A_ immunobinding in the raphe nuclei, hippocampus, substantia nigra, basal ganglia and forebrain, which remains low until PND 120. PC exposed females exhibit reduced immunobinding in the raphe throughout development with other regions normalizing by puberty and remaining normalized throughout adulthood, with the exception of the forebrain, which shows a surge in 5-HT_1A _binding during later adulthood (PND 120; Fig. **2**). 

##### SSRI Exposure

A single study has shown that exposure to fluoxetine (GD 13-21; 8 or 16 mg/kg/day) or venlafaxine (GD 13-21; 40 or 80 mg/kg/day) does not affect the response of adult rats to 5-HT_1A_ agonist-induced 5-HT syndrome, suggesting that the function of these receptors is unchanged [101]. As mentioned above, since many of the differences in 5-HT_1A_ receptors are greater following PC exposure regimens with exposure beginning prior to SERT expression [50,70], it is possible that other cocaine-induced effects are responsible for the observed changes. 

#### 5.4 Summary 

Immunobinding and radioligand binding are useful techniques to study regional changes in receptor proteins, however, further studies are needed to determine whether such changes are in pre- or postsynaptic locations. Additionally, it will be important to determine whether receptor differences occur on neurons or glia since some controversy exists over cell-type specific expression [102,103].

One important discrepancy noted in the literature is the difference between length of PC exposure and its effect on adult 5-HT_1A_ receptor expression patterns. Exposure for only the last 2 weeks of gestation results in minor changes, while exposure for the entire gestational period has long-lasting effects in males, and to a lesser extent in females. This is especially apparent when immunobinding data are compressed across brain regions (Fig. **3**). This suggests that events occurring early in embryogenesis may have long-term consequences for 5-HT_1A_ receptors. Moreover, since 5-HT_1A_ expression and function are critical for a number of behaviors, the length and timing of exposures should be considered in future clinical studies. 

These data also suggest a sex-specific effect on the stress-response as a function of 5-HT_1A _receptor activity in the hypothalamus. Future studies should investigate functional changes in 5-HT_1A _receptors in different brain regions following longer PC exposure paradigms, since it is currently unclear whether earlier exposure will cause similar sex-specific effects on 5-HT_1A_ function. Since PC exposure does not appear to impact the ability of these receptors to promote release of oxytocin, prolactin, or corticosterone at any age in either sex [100], these results suggest a sensitivity of CRF releasing neurons to PC exposure, in contrast to other neuroendocrine cells of the hypothalamus. Although 5-HT may also be having a direct stimulatory effect on pituitary cells, the interactive roles of CRF and 5-HT in releasing ACTH remains unclear [104,105]. This system may be a critical point in behavioral stress response differences observed in these offspring (see **Sections C. 3.3 C.3.4 on Hormonal and Behavioral Stress Response**), and direct investigation of the CRF signaling system would be greatly informative.

Additionally, as can be seen in Fig. (**3**), the developmental age when PC exposure has effects on 5-HT_1A_ receptors throughout the brain depends on sex. Given that the 5-HT_1A_ receptor function is dynamically regulated throughout the estrous cycle [106], and this was not taken into consideration during brain collection, the control females show a great deal of variability that PC exposed females do not. The estrous cycle does not appear to be disturbed in PC exposed females [107] suggesting that they may be less sensitive to endocrine control of their 5-HT_1A _receptors. Testosterone can inhibit the function and expression of 5-HT_1A_ receptors [108,109], and although adult testosterone levels are unaffected by PC exposure, cocaine can alter testosterone incorporation into the hypothalamus, [110], potentially causing organizational effects that could impact reactivity to changes in the hormonal milieu later in life. In addition, 5-HT_1A_ receptor activity during the perinatal period is important for development of testosterone sensitivity and aggressive behavior in adulthood [111], which could be impacted by increased expression of 5-HT_1A_ receptors during the neonatal period, as has been observed in rodents [50]. It can be proposed that early cocaine-induced changes in steroid hormonal signaling may predispose 5-HT_1A_ receptors to disrupted interactions with adult hormonal signaling, an area where further research is warranted.

Alternatively, the sex differences observed may not rely solely on hormonal causes. The absence of SERT throughout development has sex-specific effects on 5-HT_1A_, specifically SERT knockout mice show decreased 5-HT_1A_ in the raphe nuclei of males and females, but in females this effect is both more pronounced and found in several other brain regions [38]. Although these data do not mimic exactly what is seen after PC exposure, PC exposure does decrease SERT during infancy [74,85] (and potentially during embryogenesis, however this is not yet known), and this decreased SERT activity may be playing an important role in the development of 5-HT_1A _signaling. Taken together, these data highlight the complexity of the developing serotonergic system.

Nonetheless, studies of the effects of prenatal SSRI exposure on 5-HT_1A_ expression and activity could be useful in deciphering the role of SERT blockade in 5-HT_1A_ developmental dysregulation, especially since prenatal SSRIs cause similar deficits in SERT expression in neonatal life as compared to PC exposure (see **Section B. 4.** **Developmental Effects on Serotonin Reuptake Sites**), which is a potential mechanism for disrupted 5-HT_1A_ expression [38]. If such studies find that prenatal antidepressant exposure does not cause disruptive effects on 5-HT_1A_ receptors similar to PC exposure, then this may warrant further investigation to understand how 5-HT_1A_ receptor regulation occurs throughout development. Additionally, such differences might help explain the lack of many behavioral deficits seen following prenatal SSRIs compared to PC exposure (see **Section C.** **Behavioral Consequences of Prenatal Cocaine Exposure**). 

Few studies have investigated how prenatal exposure to SSRI’s affects postnatal 5-HT_1A_ receptor expression or function. However, given the role of 5-HT_1A_ receptors in the development of 5-HT syndrome, and SSRIs ability to increase the likeliness of this disorder, these receptors should be investigated [69].

### 6 5-HT_2A_ RECEPTORS

5-HT_2A_ receptors, a subtype of 5-HT_2_ receptors, are distributed throughout the cortex, basal forebrain, hypothalamus, hippocampus and hindbrain [34,112]. 5-HT_2A_ receptors have been strongly implicated in psychotic behavior [113], as well as contributing to impulsive behavior, depression and anxiety [114,115]. Similar to 5-HT_1A_ receptors, 5-HT_2A_ receptors contribute to the release of several hormones into the bloodstream in response to stress, and are commonly measured using receptor binding techniques [96,98]. 

#### 6.1 Infancy 

##### PC Exposure

There are no published data on whether expression or function of 5-HT_2A_ receptors change following PC exposure in infancy. 

##### SSRI Exposure

There have been no studies investigating the effects of prenatal SSRI exposure on 5-HT_2A_ receptors in infancy.

#### 6.2 Juvenile and Adolescent Periods 

##### PC Exposure

Male rats with PC exposure (GD 13-20; 30 mg/kg/day) exhibit increased ACTH and renin release following 5-HT_2A_ stimulation in the hypothalamus as juveniles without changes in receptor density, similar to effects observed on 5-HT_1A _function [112]. There are no data regarding differences in 5-HT_2A_ receptor expression in the adolescent period following PC exposure. 

##### SSRI Exposure

Following prenatal exposure to fluoxetine (GD 13-20; 10 mg/kg/day) or amitriptyline (GD 2-21; 10 mg/kg/day), 5-HT_2A_ receptor binding and function are unaffected in the hypothalamus and cortex of juvenile male and female rats [24,116-118]. This is in contrast to increased ACTH and renin release in adolescent PC exposed males. However, longer exposure (GD 6-21; 10 mg/kg/day) to chlorimipramine, inprodole or mianserin (less specific SRIs) results in decreased 5-HT_2_ receptor binding in juvenile rats, while exposure to a different antidepressant, nomifensine (GD 6-21; 10 mg/kg/day) (DA/NE reuptake inhibitor), increases 5-HT_2_ receptor binding, [119]. Although, these studies using SRIs were not specific to 5-HT_2A_, they suggest a very complex mechanism controlling expression of this receptor.

#### 6.3 Adulthood 

##### PC Exposure

Following PC exposure (30 mg/kg/day) on GD 13-20 or on GD 1-20, both adult male and female rats showed no differences in 5-HT_2A_ receptor expression in the hindbrain, midbrain, hypothalamus or forebrain [112,120]. Receptor dynamics (production, degradation rate and half life) were also unaffected in adults following PC exposure [98], and no effect on adult basal function (to control hypothalamic hormone release) of 5-HT_2A_ receptors was observed. PC exposure in male rats can lead to enhanced desensitization of hypothalamic receptors following 2 weeks of paroxetine treatment, while not affecting receptor expression, as measured by diminished ACTH and oxytocin responses to a 5-HT_2A_ agonist [121]. Another study investigated the role of 5-HT_2_ receptors in preventing fluoxetine-induced acetylcholine release in the striatum, and found that PC exposure had no effect in males or females, indicating function and sensitivity to 5-HT for these receptors were unchanged in this brain region [107]. Taken together, these results suggest that 5-HT_2A_ receptors in general are not greatly impacted by PC exposure, with the exception of the male hypothalamus [121]. 

##### SSRI Exposure

Decreases in receptor binding are observed in adult males [24,113,117,118] and correspondingly, adult males exhibited an attenuated 5-HT_2A_-stimulated release of ACTH and renin [117]. This is in contrast to the lack of effects in adult PC exposed males. Little other work has been reported investigating the impact on other 5-HT receptor subtypes following prenatal SSRI exposure.

#### 6.4 Summary

The results of prenatal SRI exposure are interesting in comparison to those of PC exposure, as the impact is stronger during adulthood, while PC exposed adults have recovered from differences observed in the juvenile period. This suggests that differential developmental compensations may be occurring because of the multiple mechanisms of cocaine, and highlights the importance of studying different developmental time points. This hypothesis is supported by data showing opposite effects on 5-HT_2 _receptor binding following SERT blockade compared to DAT/NET blockade. Since cocaine acts on all three transporters, it may negate the effects of DAT/NET blockade and return the system to natural development by adulthood, although this remains to be determined.

### 7 5-HT_3 _Receptors

5-HT_3_ receptors, the only ionotropic serotonin receptor, have been associated with drug abuse, aggression and depression [122-124]. These receptors are expressed at the highest levels in the brainstem, but are also present at appreciable levels in the hippocampus, amygdala, caudate-putamen, and some cortical regions [34, 122-124]. Since these receptors modulate the release of acetylcholine in the forebrain; thus, stimulated acetylcholine release can be used as a metric for 5-HT_3_ activity [125].

#### 7.1 Infancy, Juvenile and Adolescent Periods 

No published studies have investigated 5-HT_3_ receptor changes following PC or SSRI exposure in infancy, juvenile or adolescent periods.

#### 7.2 Adulthood 

##### PC Exposure

To date, only a single lab has studied the effects of PC exposure on 5-HT_3_ receptor-induced acetylcholine release in the striatum of adult male and female rats [107, 125]. These studies indicated that PC exposure (40 mg/kg/day) from GD 15-21 enhances 5-HT_3_ functional inhibition of acetylcholine release in males and diestrus females, but not in proestrus females. This suggests an interaction of PC exposure with reproductive hormonal status in 5-HT_3 _regulation of acetylcholine release, a finding that should be explored further at specific developmental stages and in other critical brain regions. 

##### SSRI Exposure

There have been no investigations of the effects of prenatal SSRI exposure on 5-HT_3 _expression or function at any age. 

### 8 Summary of the effects of PC or SSRI Exposure on Serotonergic Signaling

There are many areas of investigation where no direct comparisons have yet been made between PC and prenatal SSRI exposure, or where particular developmental stages or sex differences have not yet been analyzed (see Table **2**). Some effects of PC exposure may be dependent on SERT blockade, since prenatal SSRIs mimic these effects, while others seem to require additional mechanistic actions of cocaine. It is clear, however, that PC exposure extensively affects serotonergic signaling, with disruptions in growth factors, 5-HT levels, projections, metabolism, transport and several 5-HT receptor subtypes. There are many 5-HT receptor subtypes that have not yet been investigated following PC exposure, including 5-HT_1B_, 5-HT_1C_, 5-HT_1D_, 5-HT_2B_, 5-HT_2C_, 5-HT_4_ and 5-HT_7_. Although we may expect prenatal drug exposure to alter the regulation of these receptors, this may not be the case since 5-HT receptor genes are differentially regulated by a variety of cellular mechanisms [126]. 

One major discrepancy between the preclinical and clinical data is the age of subjects. Preclinical studies have focused primarily on adolescent and adults, with very few having studied infant or juvenile development. The juvenile period represents an area of investigation that warrants comprehensive studies of serotonergic signaling, since many behavioral effects are observed during this time frame. Conversely, clinical studies have has thus far only investigated infants and children, although it is reasonable to assume that this is due to the fact that patients are only now reaching adolescence and early adulthood. From the preclinical literature it is clear that clinical studies of adult function should be performed in the future.****

Of particular note, studies of infant hypothalamic serotonergic signaling are absent and could be informative. Such studies will be critical to understanding the full impact of PC exposure on serotonergic signaling. Additionally, surprisingly little work has assessed the effect of PC or prenatal SSRI exposure on serotonergic signaling or function within the brainstem, where 5-HT neurons are located. A well-designed quantitative study of raphe 5-HT neurons would greatly enhance our understanding of serotonergic development following PC or prenatal SSRI exposures. Transcription factors such as SHH, GATA3, PET1, MASH1 are critical to the development and differentiation of these 5-HT neurons [127,128]. PC exposure does not affect SHH expression in the brainstem at the time when 5-HT neurons originate and begin to develop, suggesting that the origin and early differentiation of these neurons are likely unperturbed [129]. However, further investigation of the effects of PC and prenatal SSRI exposure on developing 5-HT neurons, as well as other important transcription and growth factors, are needed to determine whether, in fact, 5-HT neurons are indeed unaffected.

Although researchers have focused on various aspects of serotonergic signaling in the studies reviewed here, it should be noted that serotonergic signaling pathways are highly interactive with each other and with other neurotransmitter systems during development, such that direct effects of cocaine may have indirect effects on other downstream targets. This is especially evident in the interactions between 5-HT, 5-HT_1A _and SERT, as decreased function of one component, like those observed following PC exposure, can have long lasting effects others [52,130,131]. For example, SERT hypomorphs exhibit reduced adult 5-HT_1A_ signaling, an effect mimicked by PC exposure in males, which exhibit reduced SERT during infancy [50,85,130]. However, the absence of SERT throughout development decreases 5-HT_1A_ differentially in males and females [38]. Additionally, 5-HT_1A_ knockout mice show reduced SERT binding in the cortex, hippocampus and striatum as adults [132], suggesting that the low 5-HT_1A _receptor binding observed in PC exposed female infants may predict decreased SERT expression or function in adulthood. Future studies should consider these possible developmental compensations when interpreting the impact of PC and prenatal SSRI exposure. 

## C BEHAVIORAL CONSEQUENCES OF PRENATAL DRUG EXPOSURE

There is an abundance of literature documenting behavioral changes following PC exposure, which has been the subject of several earlier reviews [133-136]. Disruptions in serotonergic signaling have been tied to developmental disorders such as autism [63,137], and PC exposed children share many behavioral phenotypes with children with autism spectrum disorders, including attention deficits, aggression and impaired social behavior, and compromised communication skills [138]. It is critical to understand the functional relevance of changes following PC exposure in children and adults who may suffer from neurobiological disruptions. Since 5-HT is associated with the regulation of numerous behaviors, behavioral changes associated with PC and SSRI exposure that may be related to functional changes in the serotonergic system are summarized below.

### 1 Attention Problems

Consistently occurring behavioral problems that have emerged from studies on PC exposure are attention/arousal deficits that are evident throughout development [139,140]. Attention in infants is typically measured by monitoring eye-gaze and corresponding heart rate changes during specific stimuli presentation [141,142]. Attention-deficit hyperactivity disorder (ADHD) is clinically defined by exhibiting inattention (difficulty sustaining attention and mental effort, forgetfulness, and distractibility); hyperactivity (fidgeting, excessive talking, and restlessness); and impulsivity (difficulty waiting one’s turn and frequent interruption of others) [143]. Preclinical models investigating the impact of PC and prenatal SSRI exposure on attention have primarily used the spontaneous alteration test as a measure of working memory and attention; although, the testing protocol, age of the test subject, and motivational factors can play important roles in the interpretation of this task [144].

#### 1.1 Infancy and Childhood

##### PC Exposure

Infants and young children through primary school with PC exposure display decreased regulation of arousal-mediated attention and behavioral state [142,145-148]. PC exposed children and adolescents, particularly males, have a higher incidence of developing ADHD [133,149]. Along with attention deficits, these children have other difficulties at school, such as abnormal externalizing behavior (e.g., acting negatively on the external environment) and oppositional defiant disorder (e.g. not cooperating with authority figures) associated with their ADHD [140,149,150]. Recent neuroimaging data suggest that PC exposed adolescents exhibit differential activation of attention/arousal regulation circuitry, including working memory neurocircuitry (specifically prefrontal cortex and amygdala), in response to distracting emotional stimuli when compared to non-PC exposed adolescents [151,152]. Although very little work has been done using animal models for this age, PC exposure (GD 4-18; 10 mg/kg/day) was found to cause significant differences in spontaneous alteration tests ( a measure of working memory and attention) in male and female adolescent rats [153]. However, a higher dose of PC exposure (GD 1-20; 30 mg/kg/day) had no effect on spontaneous alternation at PND 30 [154].

##### SSRI Exposure

Prenatal exposure to antidepressants (fluoxetine, paroxetine, sertraline) results in tremulousness, decreased arousal, sleep disturbances and differences in behavioral state regulation in newborns [155], but do not appear to affect mental development, attention, or impulsivity in young children [26,27,156]. In rodents exposed to fluoxetine at any dose (2-12 mg/kg/day) from GD 7-20, no effect was observed during the juvenile or adolescent periods on spontaneous alternation, a test of working memory [157]. However, prenatal exposure to bupropion, an antidepressant more selective for the dopamine transporter, did increase likelihood to develop ADHD [156].

#### 1.2 Adulthood 

##### PC Exposure

PC exposure during the last 2 weeks of gestation (1-6 mg/kg/day) results in decreased selective attention in adult male, but not female rats [158-160] with the exception of aged female rats who do exhibit attention deficits [161]. Interestingly, similar to adolescents, adult male rats with PC exposure (GD 1-20; 30 mg/kg/day) show no differences in spontaneous alteration [154]. Studies in clinical populations of PC exposed adults are sparse, but childhood ADHD has been associated with adult ADHD, anti-social behavior and aggression [162,163], and therefore it will be important to continue to monitor the mental health of PC exposed children diagnosed with ADHD.

##### SSRI Exposure

Similar to juvenile and adolescent rats exposed to fluoxetine at any dose (2-12 mg/kg/day) from GD 7-20, there was no effect during adulthood on spontaneous alternation [157]. Data has not been reported in clinical populations of prenatally SSRI-exposed adults. However, such data should be collected to determine if problems develop with age.

#### 1.3 Serotonergic Effects May Underlie Attention Deficits

The role of serotonergic function in attention has been studied extensively [164]. Postsynaptic 5-HT receptors in the prefrontal cortex are thought to play an important role [165]. Recent evidence also suggests a role for presynaptic 5-HT receptors in some of the symptoms associated with ADHD [166]. The 5-HT_1A_ receptor is a likely candidate for this targeted effect, since it is expressed in the attention-regulation region of the superior colliculus and is known to be expressed presynaptically [34,112,167]. 5-HT and DA interactions play important roles in regulating attention processing in several brain regions [164,165,168]. ADHD has been linked to changes in DA receptor signaling [169], and PC exposure is associated with attention deficits mediated by DA and NE signaling pathways [139,170]. In addition, difficulties in attention regulation are often co-morbid with behavioral problems in aggression or depression [162,171], both of which involve serotonergic signaling and are disrupted by PC exposure (See **Section C. 2.** **Anxiety and Depressive-Like Behavior and Section C.5 Aggression**).

These data suggest that attention deficits following PC exposure may not be related solely to SERT blockade, but may also occur through the dopaminergic system, mediated by either blockade of the dopamine transporter (DAT) and/or SERT [39], or by effects on dopamine release caused by changes in serotonergic signaling at presynaptic 5-HT_1A_ receptors [172]. PC exposure appears to impact 5-HT_1A_ receptors, specifically decreasing 5-HT_1A_ in both the raphe nuclei and frontal cortex of male rats [50], suggesting a possible mechanism of action for attention problems in PC exposed males. Thus, 5-HT_1A_ receptor ligands might be useful tools to use with attention deficits resulting from PC exposure. Although these are important first steps in investigating how prenatal SERT blockade may contribute to attention problems, further work is necessary to fully conclude that negative consequences follow prenatal exposure and to investigate potential mechanisms.

### 2 Anxiety and Depressive-Like Behavior

Mood disruptions including anxiety and depression are clinically defined by physicians following personal interviews and specialized questionnaires such as the Hamilton Rating Scale for Depression (HAM-D) or Anxiety (HAM-A) [173,174]. Consistent and reliable tests have been developed to assess anxiety and depressive-like behavior in rodent models. These include the elevated plus maze, open field test, light-dark box and social interaction for anxiety [175,176]. Measuring a depressive-like state in the rodent is routinely studied using a forced swim test, tail suspension test for assessing a “despair” phenotype, or by measuring the animal’s motivation for hedonic stimuli [177]. Unfortunately, there are no validated tests for anxiety or depressive-like behavior in rodent infants (younger than PND10), since ultrasonic vocalizations (USVs) have been suggested to be largely a function of thermogenesis regulation and not anxiety in infant rats at this early age [178,179], although these are communicative sounds and will be discussed later (see **Section C.7.** **Language and Communicative Ability**). However, in juvenile rats and mice, separation-induced USVs are commonly used as a marker for anxiety, given that they can be decreased by anxiolytics [180,181].

#### 2.1 Infancy and the Juvenile Period 

##### PC Exposure

Little is known about the impact of PC exposure on the development of anxiety or depressive behavior during infancy or childhood in humans. Given that PC exposure increases the likelihood of developing ADHD and aggression, and that these behavioral problems are often co-morbid with mood disorders, future clinical studies should measure anxiety in children following PC exposure [162,163,182]. Several recent preliminary studies have found altered USVs from PC exposed rats at PND 14 [183,184]. Following PC exposure (GD 1-20; 30 mg/kg/day), both male and female juvenile rats have been shown to exhibit enhanced anxiety-like behavior in the open field [185,186]. The same treatment paradigm also results in neophobia (as measured by hypoactivity in a novel environment compared to controls) between PND 20 and 35 [153,187,188]. Interestingly, intermittent PC exposure (30 mg/kg/day) throughout pregnancy, to model the “weekend user,” showed no effects on locomotor activity or anxiety-like behavior [187], indicating that amount and frequency of exposure may be important variables.

##### SSRI Exposure

Children exposed to antidepressants (fluoxetine, paroxetine, or sertraline) do not show major differences in anxiety or depressive symptoms according to the Child Behavior Checklist [189]. Male and female juvenile mice show increased separation-induced USVs following full gestational exposure to the SSRI paroxetine (30 mg/kg/day), suggesting increased anxiety [190]. Several studies have shown that treatment with either paroxetine or fluoxetine does not affect elevated plus maze behavior (anxiety-like behavior), forced swim (depressive-like behavior) or general locomotor activity in male adolescent mice or rats [25,28,157,190]. In male rats, prenatal exposure to the less specific SRI, chloripramine (GD 8-21; 3 or 10 mg/kg/day), results in an anxiolytic effect, as measured by the social interaction test, but an anxiogenic effect as measured by the open field test [191]. Following pre- and early postnatal fluoxetine exposure (GD 1-PND 14; 7.5 mg/kg/day), adolescent female mice exhibit increased depressive-like behavior in the forced swim test [25], suggesting that females are more sensitive to SSRI exposure. 

#### 2.2 Adulthood 

##### PC Exposure

Adult male and female PC exposed (GD 1-20; 30 mg/kg/day) rats exhibit increased depressive-like behavior when tested at PND 60 and 120, while exposure to a higher dose (GD 8-20; 40 mg/kg/day) results in expression of depressive-like behavior in adults for up to 12 months [192]. The literature concerning anxiety-like behavior in rodents is less clear. PC exposure (GD 8-20; 30 mg/kg/day) increases anxiety-like behavior in the elevated plus maze and the open field test at PND 60, with continued increases up to one year in male and female rats [161,192,193]. In contrast, PC exposure (GD 1-20; 30 mg/kg/day), either chronic or intermittent, only has a minor impact on anxiety-like behavior as measured by elevated plus maze, open field or social interaction at PND 60 in males or females. However, severe neophobia was observed at this age, which was more pronounced following chronic PC exposure compared to intermittent PC exposure [154,185,187]. By PND 90, a trend towards neophobia was still observed, with no other effects in males, although females showed slightly less anxiety on the elevated plus maze [153,154]. By PND 120, anxiety returned in males, as measured by social interaction, but not elevated plus maze [185]. In contrast, following intermittent PC exposure, only males showed hyperactivity in the open field [154]. Taken together, these results suggest that PC exposure can affect anxiety-like behavior depending on sex and age of testing. A future focus on these behaviors in human populations might prove useful, since anxiety is highly correlated with ADHD [194], and such children are at high risk for this disorder, as discussed above. 

##### SSRI Exposure

Fewer studies have investigated the effects of prenatal SSRI exposure on later emotional regulation. Similar to adolescents, adult male mice show no effect of prenatal paroxetine (GD 1-21; 30 mg/kg/day) treatment on anxiety or depressive-like behavior [190], while exposure to fluoxetine (GD 1-PND14; 7.5 mg/kg/day) increases depressive-like behavior in female, but not male mice [25]. Prenatal exposure to the less specific SRI, chloripramine (GD 8-21; 3 or 10 mg/kg/day) had no effect on anxiety, as measured by the social interaction test or open field test, in male rats [191]. These data suggest that females may be more susceptible to the effects of prenatal SSRI exposure on emotional behavior throughout development.

#### 2.3 Serotonergic Signaling Involvement in Anxiety and Depression

Anxiety and depression are correlated with 5-HT levels, SERT, 5-HT_1A_, and 5-HT_3_ receptor activity [80,122,195,196], all of which are also disrupted in PC exposed male offspring. Serotonergic changes may underlie differences in depressive-like behavior observed in both male and female PC exposed offspring. This could be directly tested using pharmacologic methods to determine systemic changes that result in specific behavioral effects. Anxiety and depressive behavior have also been strongly tied to stress reactivity [197,198]. Therefore, it is possible that these behaviors are related to alterations in stress response systems that are often seen in PC exposed offspring (see **Sections C.3. and C.4 Hormonal and Behavioral Stress Response**). These results suggest that females are especially sensitive to prenatal SSRI exposure compared to males, although differences in SERT expression or activity, which may be expected with differences in depressive behavior, have not been investigated in females. Future preclinical studies could investigate the impact of prenatal SSRIs on female 5-HT levels and terminals, which might also be involved in increased depressive behavior in females. Evidence suggests that prenatal SERT blockade may be sufficient to cause changes in female behavior, while other mechanisms of cocaine action may be more critical for effects on male behavior and neuronal function. However, blockade of the DAT alone may not be sufficient to cause changes in male behavior, since studies with prenatal exposure to amfonelic acid (AFA), a drug that blocks the DAT, causes only minor changes in spontaneous activity but not anxiety-like behavior, at least during adolescence [154,187]. AFA exposure did result in hypoactivity, and a tendency to exhibit neophobia by PND 60, but not PND 180, in males [154,187]. The antidepressant, bupropion (primarily a DA transporter blocker, with greater affinity for SERT than AFA; see Table **1**), can cause increased anxiety in adult male mice [199] similar to that observed with PC exposure, suggesting that disruptions in these signaling systems are complex, but still potentially important for the development of anxiety.

### 3 Hormonal Stress Response

Response to physiological or psychological stressors involves activation of the hypothalamic-pituitary-adrenal (HPA) axis to release corticotrophin releasing factor (CRF), adrenocorticotrophic hormone (ACTH) and cortisol (corticosterone in rodents or CORT). Typically, this response begins immediately and causes hormone levels to peak five to 10 minutes after the stimulus, followed by a return to baseline levels within an hour. Although it is extremely adaptive to exhibit a hormonal stress response, higher levels for longer periods of time in a test group indicate differences in stress perception, and can have long-lasting effects on physiology [200]. Importantly, CRF is released from the paraventricular nucleus (PVN) of the hypothalamus, and acts as a hormone in the bloodstream and as a neurotransmitter throughout the CNS, including the limbic system, providing an avenue for stress signaling to interact with other behaviors, including anxiety, depression and aggression [197,201]. Although hormonal and behavioral stress responses are generally congruent, it has been shown that CRF can act in the forebrain to produce a behavioral response independent of a hormonal response, suggesting that regional expression of CRF is critical for appropriate integrated responding [202]. One important aspect of PC exposure that has been noted is a differential response to stressful stimuli in males and females [136,203]. 

#### 3.1 Infancy and the Juvenile Period 

##### PC Exposure

Studies in human PC exposed infants indicate that age of testing is important for interpreting results. One study found that PC exposed infants show no difference in basal CORT and lower CORT in response to both invasive and non-invasive procedures at 2 months [204], whereas another study found lower basal CORT levels but no difference in response to a pin prick 13-months [205]. Recently in PC exposed 11 year olds, blunted CORT response to a psychological stressor was observed, with no differences in baseline CORT, mimicking the results of the 2-month old study [206]. These results suggest either a developmental compensatory response or a reaction to the hormonal milieu. These changes may also interact with the early life environment (See **Section D. 1.1 Maternal Drug Use Disrupts Maternal-Infant Interactions**). PC exposed (GD 8-20; 40 mg/kg/day) rats exhibit higher CORT and ACTH levels, with correspondingly higher c-FOS expression in the PVN of the hypothalamus (where CRF is produced) in response to acute and repeated foot shock-induced stress, when tested as juveniles [207]. Basal levels of ACTH and CORT are unchanged during adolescence in PC exposed (GD 13-20; 30 mg/kg/day) animals, although males have an increased ACTH response to 5-HT receptor agonists, while females have a diminished response compared to controls [24,112,208].**Additionally, at PND 30, PC exposed (GD 1-20; 30 mg/kg/day) male rats show an extended and greater rise in ACTH and CORT to restraint stress [209]. These results suggest that regardless of exposure length, males have increased HPA activity following PC exposure. 

##### SSRI Exposure

Less is known regarding the effects of prenatal exposure to SSRIs on the hormonal stress response in humans. A recent study found that human infants prenatally exposed to SSRIs (fluoxetine, paroxetine, sertraline, citalopram, or venlafaxine) had reduced basal CORT levels, and although in control infants, breast-feeding infants differed from bottle-feeding infants in their basal CORT levels, SSRI exposed infants did not differ based on feeding type [173]. These data suggest that these infants may be less responsive to neonatal environments. 

#### 3.2 Adulthood

##### PC Exposure

In preclinical studies of PC exposure, basal levels of stress hormones do not differ from controls at adulthood [112,209]. However, several studies have obtained disparate results regarding the impact on the HPA axis in response to stressful stimuli; while some studies (GD 8-17: 20; 40 mg/kg/day) found no change [210], others found an increased [209,211] or decreased response [186]. Specifically, PC exposed (GD 1-20; 30 mg/kg/day) males show lower ACTH, but not CORT 90 min after an elevated plus maze and following exposure to a stranger male, although females show no difference due to PC exposure [186]. These conflicting results, suggesting either increased reactivity following PC exposure or an inappropriately blunted response, probably depend on the type of stressor, timing of hormonal assay, hormones measured, and extent of PC exposure. 

##### SSRI Exposure

We could find no studies investigating stress reactivity at later time points in either clinical or preclinical models. This will be an important area for future study, not only to understand long term effects of prenatal SSRI exposure on stress reactivity, but also for understanding the role SERT blockade plays in mediating the consequences of PC exposure.

#### 3.3 Changes in Serotonergic Signaling May Underlie Stress Responsiveness

The important interactions between canonical stress hormones and serotonergic signaling may be central to understanding some of the hormonal effects observed following PC exposure. CRF interacts with serotonergic mechanisms in several ways [212]. 5-HT_1A_ activity in both the raphe nuclei and the PVN can drive ACTH secretion, suggesting an indirect increase in CRF activity [213]. Alternatively, SERT activity can block CRF-induced behavioral responses [214]. Given that 5-HT_1A_-induced release of ACTH is increased in PC exposed males, but not females, this effect may be functionally related to male-specific changes. The potential importance of 5-HT_1A_ receptors in male responsivity is strengthened by lack of evidence for changes in SERT activity in adulthood following PC exposure (See **Section B.4.3 Developmental Effects on Serotonin Reuptake Sites**). CRF expression and activity following PC exposure has yet to be measured, but would be highly informative, since it: 1) can drive the observed ACTH changes; 2) can play a critical role in stress reactive behaviors (anxiety and depression); and 3) is active in brain regions where decreases in 5-HT_1A_ receptors may play important roles [50,198,208]. Differences in regional CRF expression may potentially explain sex differences in behavioral stress response and anxiety observed following PC exposure. Future studies that focus on developmental differences in CRF activity or receptor expression following PC exposure to determine if they correspond with changes in serotonergic signaling, as well as directly test involvement of the HPA regulatory feedback system to determine the exact deficits responsible for the observed behavioral changes in stress response, would be useful.

### 4 Behavioral Stress Response

Behavioral stress response is commonly defined as any behavior exhibited immediately following the presence of stressful stimuli. Stressful stimuli are either inferred by the investigator or have been previously shown to increase hormonal stress levels. In children and adults, this may include crying, facial expressions of displeasure and signs of irritability. In animal models, changes in locomotor activity (including freezing), ability to habituate to novel or fearful environments and expression of USVs are also interpreted as behavioral responses to stress. Additionally, neuronal activation studies have begun to determine brain circuitry activity that may inform the literature about the perception of these stimuli.

#### 4.1 Infancy and Childhood

##### PC Exposure

PC exposed human 7 month old infant boys and girls exhibit a decreased latency to anger during an infant restraint reactivity test [215]. PC exposed human toddlers show a greater arousal to a frustrating task compared to non-exposed children, with males being particularly sensitive to the test, as indicated by increased requests for the caregiver [216]. This is especially interesting given that another study found PC exposed toddlers showed less negative reactivity to separation from their mothers, typically considered a stressful event [217]. PC exposed children at age 5 continue to exhibit a decreased latency to frustration, with males showing the strongest effect [218]. Following PC exposure (GD 8-20; 40 mg/kg/day), juvenile male rats exhibit shock-induced hyperactivity and less wall-climbing, indicating a direct response to the stressful environment [219]. Following the same PC exposure, juvenile male, but not female, rats retain the habituated orientation response longer than controls, suggesting that they react differently to this stressful environment [220]. 

##### SSRI Exposure

Very little work has been done to study the effects of prenatal SSRI exposure on behavioral stress response in young children. However, one study has shown children between 15 and 30 months who were prenatally exposed to fluoxetine or tricyclic antidepressants do not show any differences in temperament or other stress responsive behaviors [221]. Externalizing behaviors (acting negatively on the environment) do not seem to be increased by prenatal SSRI exposure, but instead depend on maternal depression [26]. These data suggest that prenatal SSRI exposure does not negatively affect behavioral stress response in infants or young children, however, more specific testing should be performed before final conclusions are drawn.

#### 4.2 Adolescence 

##### PC Exposure

In one study, both male and female adolescent rats showed less habituation to a repeated stressor following PC exposure (GD 8-21; 40 mg/kg/day) [222]. Although amygdala neuronal activation to footshock seemed unchanged, cortical response was increased in adolescent PC exposed (GD 10-20; 6 mg/kg/day) male rats, suggesting an altered perception of a normally stressful stimulus. Unfortunately, females were not tested in this study [223]. 

##### SSRI Exposure

There are not yet any studies investigating the affect of prenatal SSRIs on adolescent behavioral stress response.

#### 4.3 Adulthood 

##### PC Exposure

Male rats with high-dose PC exposure (GD 8-20; 80 mg/kg/day) show greater struggling during a forced swim test, which has been interpreted as difficulty coping with a stressful situation [224]. Mice with PC exposure (GD 9-17; 20 and 40 mg/kg/day) exhibit a shorter latency to freeze following footshock [210]. Similarly, rats with lower-dose PC exposure (GD 8-20; 40mg/day) showed less behavioral adaptation to a stressful footshock [225,226], with increased cortical response but unchanged amygdala neuronal activation [223], suggesting that additional circuitry may have been activated and that pain perception is very different in these animals. PC exposed female rats (GD 1-20; 30 mg/kg/day) produced more USVs compared to controls in response to an air puff at PND 180, again indicating that there is a difference in stress response to sensory stimuli [227].

##### SSRI Exposure

Although the literature on behavioral stress response following prenatal SSRI exposure is sparse, a few recent studies have shown that prenatal exposure to the antidepressant bupropion (a NE and DA transport blocker; GD1-20; 25 mg/kg/day), but not citalopram (GD1-20; 5 mg/kg/day), a SSRI, causes increased stress susceptibility (as measured by anxiety and vulnerability to drug seeking) in adult male mice [199,228]. These data suggest that prenatal SERT inhibition may not be the primary factor in disrupting stress reactivity.

#### 4.4 Changes in Serotonin may Underlie Changes in Behavioral Stress Response

Certain changes in infant behavior following PC exposure are similar to those observed with 5-HT syndrome, including tremulousness, restlessness and well-documented effects on sleep [153,229-231], although no studies have been performed to assess this connection directly. However, PC exposure causes sex-specific changes in 5-HT_1A_ receptors in the hindbrain (see **Section B. 5. 5-HT_1A_ Receptors**), which are believed to contribute to the behavioral symptoms of 5-HT syndrome [69], suggesting that hindbrain 5-HT signaling may underlie some neonatal behavioral differences and indicating future studies should include investigations of sex differences in neonates.

These data suggest that, although further work is needed, prenatal SERT blockade alone may not be enough to cause changes in behavioral stress response, since there are many more effects observed in PC exposed children and offspring. Behavioral response to stress relies on mood state (anxiety and depression) and learning from previous experiences (such as aggressive interactions). Since these other behavioral changes are more greatly affected by PC exposure compared to SSRI exposure, they may contribute to the severity of changes in behavioral stress response observed thus far. Alternatively, fewer studies have been performed to examine this arena, especially in females, and such studies would be greatly informative. 

### 5 Aggression

Aggression is broadly defined as any action meant to cause harm to another individual, usually preceded by a conflict, although initiation of conflict is also considered aggressive. Studies of human aggression typically operationally define the definition, although are commonly divided into instrumental and reactive aggression [232]. Both, however, commonly measure violence, hostility and retaliation [233]. Instrumental aggression is a goal-driven action, while reactive aggression is a response to the current environment [232]. Childhood aggression is typically reported using the Childhood Behavior Checklist [233]. Instrumental aggression is not commonly observed in young children but increases during adolescence, while reactive aggression can be observed earlier [232]. Aggressive behaviors in rodents fall into 2 major categories: offensive and defensive. Offensive behaviors are analogous to instrumental aggression in humans, serving primarily to obtain resources. Defensive behaviors are analogous to reactive aggression in humans, serving to protect one’s self, young or territory from harm [232,234].

#### 5.1 Childhood and Adolescence

##### PC Exposure

In human children, it has been noted that PC exposure is correlated with increased aggressiveness in males, an effect that has not yet been observed in females [235,236]. Studies targeting aggression in older children and adolescents are not yet available, as they are currently being tracked. Hopefully, future studies will reveal the longer-term impact of PC exposure on aggressive behavior in humans, especially since externalizing behavior, more common in PC exposed children [149], is associated with aggression in adulthood [237]. PC exposed (GD 8-20; 40 mg/kg/day) juvenile rats have a lower latency, but a shorter duration, of nipple attachment during a nipple competition task, a test for early social dominance [238]. Similar PC exposure results in adolescent males, but not females, which are significantly more aggressive in a social competition for water following water deprivation [238]. However, following the same PC exposure (40 mg/kg; GD 8-20), adolescent males were no different or performed fewer pouncing or pinning behaviors compared to controls during a play behavior task, although females were not measured in this study [222]. These results suggest an interaction of stress on aggressive behavior, since the aggression is more notable following the stress of overnight water deprivation.

##### SSRI Exposure

There are, as yet, no clinical studies regarding effects of prenatal SSRI exposure on aggressive behavior, although given the prevalence of aggressive disorders in PC exposure and the impact prenatal SSRIs have on adult aggression (see **Section C. 5.2**.), children prenatally exposed to SSRIs should be monitored closely. 

#### 5.2 Adulthood 

##### PC Exposure

PC exposure (GD 13-20; 25 mg/kg/day) increases aggression in adult mice [239]. Similarly, adult male PC exposed (GD 8-20; 40 mg/kg; GD 8-20) rats show increased aggression in a water competition task [238]. Not surprisingly, longer PC exposure (GD 1-20; 30mg/kg/day) result in males that are more aggressive and defensive in early and later adulthood to intruder males [154,186]. Additionally, intermittent PC exposure (GD 1-20; 30/mg/ day every fifth day) also results in increased aggressive behavior towards an intruder male [240]. PC exposed males also fail to show the decrease in defensive behavior observed in controls following a geprione (5-HT_1A_ agonist) injection [240]. Interestingly, PC exposed females (GD 1-20; 30 mg/kg/day) also exhibit increased aggression in both social interactions with other females [186], and maternal aggression directed at intruder males, indicating effects that are maintained through adulthood and through the major hormonal changes associated with pregnancy and lactation [21].

##### SSRI Exposure

Animal studies that have investigated how prenatal SSRI exposure impacts aggression have shown increases in aggression similar to those observed following PC exposure. Chronic prenatal SSRI exposure to either fluoxetine (GD 1-PND 14; 7 mg/kg/day and GD 13-21; 10 mg/kg/day) or paroxetine (GD 0-21; 30 mg/kg/day) increases aggressive behavior in adult male mice and rats without changing this behavior in females [25,190,241], suggesting that fetal SERT inhibition may differentially impact male and female aggressive behavior. Rats with prenatal AFA exposure have shown increased aggressive behavior compared to controls following a 5-HT_1A_ agonist, gepirone, but not under basal conditions [240]. Future studies should examine earlier time points and different prenatal exposure paradigms to confirm the role of SERT inhibition in the development of increased male aggressive behavior. 

#### 5.3 Changes in Serotonergic Signaling May Underlie Increased Aggression

**Fig. (4):**
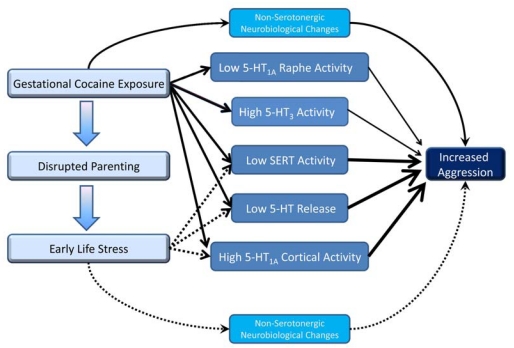
PC exposure results in increased aggression through both serotonergic and non-serotonergic neurobiological changes as indicated by the solid lines. Early life stress can also increase aggression through changes in both serotonergic and non-serotonergic signaling components as indicated by the dotted lines. If an animal experiences both PC exposure and early life stress, the impact of low SERT, low 5-HT and high cortical 5-HT_1A_ may be even greater than either insult alone contributing to a greater behavioral impact as indicated by the thicker arrows. It is likely that PC exposure offspring would experience early life stress through parental neglect induced by drug use by the parents as indicated by the blue arrows.

Changes in serotonergic signaling resulting from PC exposure may underlie some of the changes in aggressive behavior discussed above. Increased serotonergic tone has an overall inhibitory effect on aggression in preclinical studies [242,243]. A recent review of these studies highlights important factors to be considered, including: the species and strain of rodent utilized, which impacts genetic predisposition; and different treatment paradigms of serotonergic manipulations, given the importance of the dynamic interplay of serotonergic signaling systems in aggressive behaviors [244]. PC exposure in rodents results in lower basal 5-HT, disrupted 5-HT_1A_ and SERT activity, and increased 5-HT_3_ activity, all of which could lead to more impulsive or aggressive behavior in offspring (see Figs. **3** and **4**). Specifically, low basal levels of 5-HT are correlated with increased aggression [79], an effect that is observed in many brain regions in PC exposure animal models in both males and females (See **Section B.2.** **Serotonin Levels and Neuronal Growth Throughout Life**). 5-HT_3_ activity, particularly in the prefrontal cortex, nucleus accumbens and medial amygdala, has been positively associated with aggression [124,245], and increased 5-HT_3 _activity in the striatum has been observed in PC exposed mice [107,125]. Although both adult male and female rats show increased aggression following PC exposure, these behavioral changes may be accounted for by different mechanisms.

Importantly, with respect to the results discussed above, stimulation of frontal cortical 5-HT_1A_ receptors acutely reduces aggression. Consistent with this, PC exposed male rats have lower 5-HT_1A_ binding, suggesting lower basal 5-HT_1A_ activity [95,113,246-248]. Moreover, PC exposed males are less sensitive to geprione to decrease defensive behaviors, suggesting that 5-HT_1A_ receptors are less able to regulate aggressive interactions in PC exposed males [240]. Additionally, testosterone has been shown to interact with 5-HT_1A_ receptors during the neonatal period to influence offensive and defensive behavior in adults [111], and PC exposed males have increased 5-HT_1A_ receptors at this time (See Fig. **3**), possibly potentiating this effect. 

Alternatively, PC exposed females, have increased 5-HT_1A_ receptor binding specifically in the forebrain during adulthood (See Fig. **2**), a time point when aggression is increased (See **Section C.5.2 Aggression**) [21]. These findings mirror results in hamsters bred for highly aggressive behavior which have higher numbers of 5-HT_1A_ receptors in the anterior hypothalamus, lateral septum, nucleus accumbens and prefrontal cortex [124]. This is consistent with the fact that in humans, increased 5-HT_1A_ receptor binding in prefrontal cortex is correlated with higher aggressive behavior [246], illustrating the importance of regional localization of these receptors for their role in aggression and sex-specific differences in serotonergic regulation of aggression. 

It is important to note that increased 5-HT_1A_ receptor activity has been directly related to decreased 5-HT release [34,249-251] in rat models, which may explain the lower 5-HT levels seen in PC exposed rats (See **Section B.2** **Serotonin Levels and Neuronal Growth Throughout Life**). However, immunobinding data indicates fewer 5-HT_1A _receptors in the raphe nuclei in both adult males and females (see Fig. **2**), suggesting that the activity of these receptors may have undergone compensatory functional changes to influence 5-HT levels or that changes in 5-HT_1A_ receptor during infancy may have had long-lasting effects on 5-HT production. Future studies investigating the direct role of these changes in 5-HT signaling mechanisms and their role in aggression will be greatly informative. In addition, disrupted 5-HT release in conjunction with increased or altered 5-HT_3_ and/or 5-HT_1A_ activity contribute to increased levels of aggressive behavior differently across developmental stages. Taken together, these findings raise the possibility of pharmacologic intervention with serotonergic drugs, which may prove useful in treatment of aggression in PC and prenatal SSRI exposed individuals. 

### 6 Social Behavior

Social behavior is broadly defined as any behavioral interaction with another individual from the same species. In humans, social behavior necessitates some form of communication through either verbal or body language, including eye contact. We focus here on the positive social interactions, excluding aggression, which are rewarding to humans and animals [252,253]. The study of social interaction in clinical and preclinical studies usually entails behavioral analysis of interaction including approach/avoidance, active investigation of individual, and the amount and type of physical contact [254]. During infancy, social interaction refers to behavioral episodes that are not specifically related to infant health and well-being. Social interactions increase in juvenile and adolescent humans and rodents, with selection for interaction with their own peer group, followed by a decline as adulthood is reached [253,255,256]. Understanding social interaction includes studies that investigate brain structures involved in processing social information/cues and performing social interactions. 

#### 6.1 Infancy and the Juvenile Period 

##### PC Exposure

Both male and female PC exposed human infants differ from non-exposed infants in their response to the Still-Face task, a measure of interpreting social cues [257], and young children with PC exposure have higher rates of autistic spectrum disorders and deficits in social and play interactions [258]. PC exposed toddlers show less reactivity to separation from their mothers [217]. Additionally, PC exposed children are less empathic and exhibit greater frontal cortical asymmetric activity when shown a crying infant or their own mother [259], supporting the hypothesis that frontal cortex function is critical for such behavioral disruptions. PC exposed children have a higher rate of oppositional defiant disorder, another indicator of misunderstanding appropriate social context [149]. PC exposed (GD 1-20; 30 mg/kg/day) infant rats are less capable of eliciting maternal care from normal rat mothers [260]. Social context can influence physiological response to stress (e.g., increased heart rate) in control juvenile rats, although in PC exposed (GD 8-20; 40 mg/kg/day) animals this effect is not present, indicating that social context is perceived differently [261]. Therefore, it is important to repeat a similar study in PC exposed children to determine whether physiological differences in response to different social contexts are apparent. 

##### SSRI Exposure

The only study to date that has investigated gestational exposure to the SSRI paroxetine (GD1-21; 30 mg/kg/day) found no differences in juvenile male and female rat social play behavior [190]. 

#### 6.2 Adolescence 

##### PC Exposure

PC exposed (GD 1-20; 30 mg/kg/day) male and female rats spend less time interacting with novel rats at PND 30 [185]. Furthermore, although both male and female adolescent rats show no difference in initiated play behavior following PC exposure (GD 8-21; 40 mg/kg/day), they are less capable of eliciting social interaction from age-matched controls [222,262]. PC exposure (GD 1-20; 30 mg/kg/day) does not, however, have an effect on pup-induced maternal behaviors (concaveation) in early adolescent male or female rats [263], a task where there is no direct elicitation from the pups, suggesting that there may be something about PC exposed adolescent offspring that is aversive to their peers. Further studies should explore this developmental period, since it is critical for developing appropriate adult social interactions. 

##### SSRI Exposure

No studies have investigated later time points in development with respect to social interactions following prenatal SSRI exposure. Future studies to determine whether prenatal SSRI exposure affects any of these behaviors would be informative, given the numerous effects caused by PC exposure and the increases in aggression noted after prenatal SSRI exposure (see **Section C.5.1 Aggression**).

#### 6.3 Adulthood 

##### PC Exposure

In adult PC exposed (GD 13-20; 25 mg/kg/day) mice, there are higher amounts of fleeing from and avoidance of novel mice [239]. Similarly, rats exposed throughout gestation (GD 1-20; 30 mg/kg/day) show decreased duration of social interaction with unfamiliar peer rats as adults, just as they do in adolescence [185]. PC exposed males take longer to reciprocate contact at PND 90, while females take longer to allow contact and are more likely to rough groom unfamiliar female rats [186]. PC exposed males also have blunted hormonal response to exposure to a strange male, indicating an altered perception of the social environment [186]. PC exposure (GD 1-20; 30 mg/kg/day) disrupts later maternal behavior exhibited by female rats [260], but does not impact pup-induced male parental behavior in rats [263].These data suggest that such deficits can last throughout life, but further studies are needed to determine whether such effects can be generalized to both sexes or to the critical adolescent period. Given the high correlation between social disorders and ADHD in PC exposed children, clinical trials might focus on infant developmental studies, with close monitoring of prenatally exposed children for specific behavioral problems [147,264]. 

##### SSRI Exposure

No studies have investigated later time points in development with respect to social interactions following prenatal SSRI exposure. Future studies to determine whether prenatal SSRI exposure affects any of these behaviors would be informative, given the numerous effects caused by PC exposure and the increases in aggression noted after prenatal SSRI exposure (see **Section C. 5.2.** **Aggression**).

#### 6.4 Changes in the Serotonergic System May Directly Impact Social Behavioral Deficits

Serotonergic involvement in regulation of social interactions remains largely unexplored. Given that low 5-HT levels in mice are associated with low sociability [265] and higher aggression [244], and that serotonergic signaling is thought to be disrupted in autism [137], it seems reasonable to hypothesize there is a role for 5-HT in these behaviors. Additionally, social behaviors can be disrupted if there are co-morbid problems with stress, depression or attention [266-268], behavioral disorders that also occur at an increased frequency in PC exposed children (see **Sections on C.1. Attention, C.2. Anxiety and Depressive-Like Behavior and C.3 and C.4 Stress Response)**. Of note, PC exposure has been linked to an increased likelihood of a diagnosis of autism spectrum disorder [258], along with decreasing CNS 5-HT levels (See **Section B.2. Serotonin Levels and Neuronal Growth Throughout Life**). Further work is needed to determine whether there is a direct relationship between serotonergic deficits and social behavior in PC exposed individuals. Similarly, it is difficult to determine at this time whether SERT blockade plays an important role in the social behavior deficits observed following PC exposure since there is a paucity of data surrounding fetal SERT blockade and later social behaviors. 

### 7 Language and Communicative Ability

Another especially important aspect of social behavior is communication between individuals. Although a variety of ways to communicate exist, humans have the unique advantage of language (verbal and visual) to express our thoughts and feelings. Language and its development is a well developed field of study too broad to cover here. Studies on language development may focus on vocabulary and grammatical structure, as well as the type of language typically used by an individual. Expressive language is the ability to produce language, while receptive language is the ability to understand language, and pragmatic language is the ability to integrate a variety of language cues and effectively use language in a social environment [269]. Importantly, recent research has shown that the neural correlates of language may exist at birth, and that social interaction increases language learning in infants [270]. Ultrasonic vocalizations (USVs) constitute one major method of communication for rodents, including pups [251,252]. Rodent models of vocalized communication have proven useful for understanding social, emotional and stress responding in adults [263-266]. Therefore, understanding changes in rodent USVs may help us understand human language.

#### 7.1 Infancy and Childhood 

##### PC Exposure

PC exposure disrupts human newborn vocalizations [146], which are important signals to caregivers. PC exposure results in well documented disruptions in language development in early childhood, especially expressive compared to receptive language [135,258,271-275], although this is not always the case [276,277]. Differences in expressive versus receptive language abilities are dependent on age and sex [272,273], such that males are generally more greatly affected by PC exposure. Future studies should continue to investigate whether these problems persist throughout adulthood. Differences in the ability to communicate normally could impact social interactions with peers throughout life, as well as potentially contributing to aggressive behavior [278]. 

Ultrasonic vocalizations (USVs) constitute one major method of communication for rodent pups [279,280]. Several studies have reported alterations in USV following prenatal drug exposure [281-285], although results following PC exposure have been mixed [40]. Neonatal exposure to cocaine (PND 4-11; 20 mg/kg/day) seems to cause either an increase or decrease in isolation-induced USV on PND 14 [282,283], and PC exposure (GD 7-17; 20 mg/kg/day) has been reported to decrease USV in mice [284]. Several recent studies have shown alterations in the number, mean duration, and spectral characteristics of rodent pup USVs following PC exposure (GD 1-20; 30 mg/kg/day) [183,184,286,287]. These differences may impact the type of maternal care the pups receive [287], indirectly impacting the developing serotonergic system (see **Section D.2.** **Early Environmental Stress Disrupts Serotonergic Signaling Throughout Development**).

##### SSRI Exposure

Very little work has been done to investigate differences in human communication following prenatal SSRI exposure. Infant crying is affected by prenatal exposure to antidepressants, and these effects are known to last up to 2 months after birth [33]. However, no differences have been observed in language development in 2-5 year old children [221], suggesting that changes in infant communication may be a transient effect. These data suggest that SERT inhibition may not be a critical factor in these issues in PC exposed children. 

#### 7.2 Serotonin Receptor Changes May Explain Early Deficits in USVs 

Preclinical studies can help guide clinical research to focus on receptors mediating particular behaviors. Studies investigating the role of early changes in 5-HT receptor expression in pup USVs have provided evidence that 5-HT_1A, _but not 5-HT_2A _or 5-HT_3_, receptors are important for separation–induced USVs in infant rodent pups. Full (8-OH-DPAT), partial (buspirone), and highly-selective (PRX-00023) 5-HT_1A_ receptor agonists decrease USVs [180,288,289], which is consistent with increased 5-HT_1A_ receptor expression and decreased USVs observed in PC exposed male pups. This suggests that a change in receptor expression may be responsible for these effects, although this has not been directly tested. 

These results indicate an important role for 5-HT_1A_ in the communicative ability of infants, although whether this receptor is also important later in development for rodent communication is currently unknown. Rodent models of vocalized communication have proven useful for understanding social, emotional and stress responding in adults [253,290-292]. Future studies could investigate whether prenatal drug exposure affects serotonergic regulation of these vocalizations in relation to the ability to elicit appropriate social interactions (see **Section C. 6 on Social Interactions**). This is of particular interest, since little is known about the role of 5-HT in language function in humans, and animal models may provide evidence that serotonergic regulation is important for the development of communication and social skills in humans. If 5-HT does play such a role, then potential pharmacologic interventions might be developed to treat PC exposed children who exhibit communication deficits. Such tools may also prove useful in treatment of a wider range of childhood disorders involving communication problems, such as autism spectrum disorders. 

## D PRENATAL DRUG EXPOSURE INTERACTS WITH THE EARLY ENVIRONMENT

The early home environment for infants and children plays a critical role in their physiological and psychological development. Parental care is the primary source for environmental stimuli in infant life and disruptions and/or removal from parental care is extremely stressful for both human and rodent infants. Early life stress causes a number of behavioral (see **Section D.2. Behavioral Effects of Disrupted Early Environment**) and serotonergic differences in adulthood (see **Section D.3. Early Environmental Stress Disrupts Serotonergic Signaling Throughout Development**); however, that early life stress may be caused by drug-induced deficits in parental care. Typically, parental care is studied in the mothers of the species, thus we will specifically discuss disruptions in maternal behavior (MB). 

### 1 Maternal Drug Use and Depression Disrupt Maternal-Infant Interactions

#### 1.1 Gestational Cocaine Exposure 

In the human population, PC exposed infants are known to have altered maternal-infant interactions, particularly since cocaine-abusing mothers are less responsive and more likely to show hostility toward their infants [293-297], increasing the likelihood that children of these mothers will suffer neglect and be placed in foster care [298-301]. In rats, gestational cocaine exposure (chronic or intermittent; 30 mg/kg/day) consistently leads to disrupted maternal-infant interactive behavior [53,260,302-304]. Deficits were observed in crouching (high arched-back nursing), licking/grooming the pups, and retrieving them back to the nest, which has been interpreted by some as indicative of a decrease in motivation for the dams to care for the pups [53,260,302-304]. Additionally, many women who abuse drugs during pregnancy will continue to do so during the postpartum period, and cocaine during this time is known to acutely disrupt maternal behavior in rats [304-307]. Human studies suggest that PC exposed infants that are premature or have low birth weight are physically unattractive and emit disturbing, high-pitched, and arrhythmic cries, leaving them more vulnerable to neglect [308-310]. The cues produced by rodent offspring are also known to contribute to the type of care they receive [260,311-313]. This is consistent with the effects of PC exposure in rodents, where pups exhibit fewer and shorter USVs with differing spectral characteristics [184,284,286,287], and therefore may receive less initial response from their dams compared to unexposed pups [260,287]. Maternal neglect can cause stress in children and rodent offspring, and hence can be interpreted as stressful early life environment.

#### 1.2 Gestational SSRI Exposure

Significantly less is understood about how SSRIs affect MB directly. SSRIs have been contraindicated during lactation; however, many women continue their use during this time. Although SSRIs are affective in some populations at relieving depression and increasing maternal gratification (the mother’s appreciation of the motherhood), they did not improve maternal-infant interactions at 8 weeks postpartum [314]. A single animal study has been performed investigating the effects of common antidepressants on MB, however; it thoroughly examined the dose response to both amnofelic acid (AFA), fluoxetine and their combination. AFA is a DAT blocker sometimes prescribed as an antidepressant in clinical population. When AFA is administered at a low dose (1.25 mg/kg/day) during pregnancy to rats, decreased nursing but enhanced licking behavior were observed; but higher doses (2.5 and 5 mg/kg/day) did not have major effects [315]. When fluoxetine was administered at 3 different doses, only the highest (16 mg/kg/day) affected MB by increasing investigation time (touching/sniffing behavior), with no effects observed on crouching, licking or nestbuilding [315]. When given in combination at the highest doses (AFA; 5 mg/kg/day and fluoxetine 16 mg/kg/day), deficits in crouching, licking and pup investigation were observed, mimicking those observed following cocaine exposure during pregnancy [315]. Aripiprizole, am antipsychotic which recently began to supplement antidepressant treatment regimens [316], can acutely disrupt nestbuilding and enhance nursing while leaving retrieval and licking unaffected [317].

#### 1.3 Postpartum Depression 

Although the impact of neonatal exposure to SSRIs is still uncertain, it is clear that maternal depression can have major negative impacts on maternal-infant interactions in humans and animal models. In a rodent model bred for depressive-like behavior on the forced swim test (Flinders-sensitive line), deficits have been reliably observed in retrieval behavior, but only after a stressful intervention; however, another line also bred for depressive-like behaviors (Wistar-Kyoto line) showed increased licking behavior in both the normative and stressed conditions compared to a control strain [318,319]. Importantly, stress during gestation has been shown to result in depressive-like behavior in the postpartum period and simultaneously disrupt MB [320]. In contrast, women with depressive symptoms in mid-pregnancy show decreased circulating CRH compared to non-depressed controls, but unfortunately this study did not compare women on SSRIs [321]. Nonetheless, these data support the hypothesis that pregnancy and lactation are periods with differential hormonal regulation. 

The studies that have been thus far performed are informative but point to an area in which little work has been done: studying the interaction between maternal depression and maternal SSRI use in the postpartum period on maternal-infant interactions. Although SSRIs do not seem to impair maternal behavior directly, infant exposure may impact infant behavior that can feedback to the maternal care received. Given the importance of treating depression during the postpartum period, and the controversy of using SSRIs as treatment, it may be prudent to defer to behavioral therapies. Behavioral therapies, including interpersonal psychotherapy, support groups and parental coaching, have been shown to decrease postpartum depression as well as improve maternal-infant interactions (see [322], for review). This avenue may be preferable in the future, but more work is clearly needed.

### 2 Behavioral Effects of Disrupted Early Environment

The negative impact of adverse childhood conditions has been widely studied (for recent reviews see [323,324]) and will not be detailed here, but rather the focus will be on behaviors disrupted following PC exposure. Disrupted maternal-infant interactions (or early life stress) associated with cocaine use by the mother could be playing a direct role in some of the behavioral effects observed in their children, including cognitive development, aggressive and social interactions, and behavioral stress response [21,133,260,263,325]. 

#### 2.1 Aggression 

Early life stress has been shown to clearly increase later aggression in animal models and clinical populations [326]. PC exposed children raised in foster care or by cocaine-using mothers exhibit greater aggression than children in stable environments [149,327]. Children of depressed mothers show greater externalizing behavior [26]. Goodwin and colleagues found that adult male rats reared by mothers who were gestationally exposed to cocaine showed greater footshock-induced aggression, regardless of their own PC exposure [328]. Similarly, female pups reared by cocaine-exposed dams showed increased maternal aggression as adults [21]. 

#### 2.2 Social Interactions 

Infants of cocaine-using mothers show decreased emotional engagement with their mothers at 18 months, a fact dependent on the quality of mothering rather than PC exposure [217]. Consistent with this, the caregiving environment has been found to be the largest factor in language development differences in PC and SSRI exposed children [221,277]. The type of mothering received directly relates to the type of mothering behavior rodents will themselves exhibit [329]. For example, maternal behavior in rat dams is disrupted in females reared by mothers gestationally exposed to cocaine [260]. Concaveation, the sensitization of virgin female and male rats to infant pups, is also decreased in juvenile male and female and adult male rats reared by cocaine-exposed mothers [263]. Taken together, these studies demonstrate that rearing by a cocaine-exposed mother can significantly affect later social interactive behaviors, and this effect is enhanced if infants were prenatally drug exposed as well. 

#### 2.3 Stress Response 

Children raised in adverse environments show the greatest impact of PC exposure, as measured by a blunted CORT response to psychological stress [206]. Although little work has been done to investigate stress after rearing by a cocaine-exposed mother, a large body of evidence exists demonstrating the importance of maternal care in the infant’s development of a normal stress response or HPA system (for reviews see [325,330,331]. Specifically, rats raised with low levels of maternal care show increased startle response and increased anxiety in an open field [332], and have increased HPA activation [330]. The mothering received also impacts the neurohormone oxytocin [260,329], another key modulator of the stress response system [333-335]. Children of depressed mothers show greater anxiety and depression, regardless of prenatal SSRI exposure [189]. 

### 3 Early Environmental Stress Disrupts Serotonergic Signaling Throughout Development

#### 3.1 5-HT Levels are Decreased

The role of 5-HT in the developing stress response circuitry has been extensively studied in preclinical models. Early work showed that separation from dams altered 5-HT, 5-HIAA, and 5-HT/5-HIAA ratios at ages PND 4, 10 and 16 [336]. The serotonergic system appears to be very sensitive to maternal care, since high levels of maternal care stimulates infant hippocampal 5-HT release, allowing for epigenetic regulation *via *acetylation of hippocampal glucocorticoid receptors (GR), effectively reducing their expression well into adulthood [337]. In contrast, low levels of maternal care decrease the amount of 5-HT released during infancy, which decreases the activity of Nerve Growth Factor-I (NGF-I) and CRE Response binding protein (CREB), DNA binding proteins that work to increase GR expression [338,339]. The increased stress response observed in offspring of neglectful mothers is similar to results observed following PC exposure. Therefore, it is possible that low levels of stimulated 5-HT release observed in PC exposed infants [74], in conjunction with low 5-HT release as a result of neglectful parenting, could act synergistically to have an even greater impact on GR expression, and hence stress responsiveness, in these animals. Importantly, animal models could be used to test this hypothesis in order to develop possible pharmacotherapies to restore normal stress responses in PC exposed children. Such treatments could include deacetylases, which have been shown to reverse maternal neglect-induced GR expression when given in adulthood (for review see [340]). 

#### 3.2 Disrupted Interaction Between SERT and 5-HT_1A_

Although the importance of normal patterns of parental care on neurobiological development has been known for some time [336], only recently has the interaction of rearing behavior with the serotonergic system received more attention. Peer-rearing in infant macaques, which is considered highly stressful, is associated with lower SERT activity and lower 5-HT levels in adulthood [341,342]. Early life stress can also decrease 5-HT_1A_ receptor function in the cortex and SERT expression throughout the brain in rats [343,344]. Recently, maternal separation has also been found to decrease hindbrain SERT in male, but not female, adult rats [345], suggesting that the male serotonergic system may be especially sensitive to disruptions in the early environment. These changes are surely aggravated by neglectful maternal care, which can also lower levels of 5-HT and 5-HIAA [326,346]. Conversely, increased care received (*via *experimenter handling of the pups) reduces anxiety and social avoidance in offspring of 5-HT_1A_ knockout mice. This suggests that enhanced maternal care can mitigate deficits caused by a lack of 5-HT_1A _activity [347]. Maternal depression can impact the development of the serotonergic system in human infants. Infants of women who were depressed during pregnancy have lower 5-HIAA and dopamine, but higher cortisol as newborn as compared to infants of non-depressed women [348]. Maternal depression was highly correlated with childhood emotional disturbances, especially in children with a low-activity polymorphism of SERT [349].

Taken together, these data indicate that neglect or early life stress result in behavioral and serotonergic signaling disruptions similar to those observed following PC exposure (see **Section B. 2** **Serotonergic Signaling Throughout Development**), especially low 5-HT levels, SERT activity, and disrupted 5-HT_1A_ activity. This is an important consideration for clinical populations, since most children remain with their biological parents, such that the early stressful environment may act synergistically with prenatal drug exposure to cause even greater disruptions than PC exposure alone. Fig. (**4**) illustrates how the combined effect of PC exposure and the early environment may interact to promote increased aggression, but could serve as an example of how prenatal exposure and early environment may interact to disrupt social behaviors or stress response as well. Fortunately, behavioral therapies targeted toward parents, and possibly pharmacologic interventions targeted toward children, can be implemented early that may be capable of minimizing the impact of PC exposure.

## E INCREASED VULNERABILITY TO EFFECTS OF PRENATAL COCAINE EXPOSURE

### 1 Sex-Related Vulnerability

Interestingly, across the behavioral and neurobiological spectrum reviewed here, PC exposed males appear more vulnerable than females, although one must consider that females generally have not received as much attention as males in studies over the years. Differences emerge during childhood (postnatal development in rodents) and increase throughout adulthood, indicating a developmental trajectory influenced by the interaction of PC exposure with androgen hormones and male behavior. Despite the fact that adult testosterone levels are unaffected by PC exposure, cocaine can alter testosterone incorporation into the hypothalamus [110], potentially causing organizational effects that could impact reactivity to changes in the hormonal milieu later in life. In addition, 5-HT_1A_ receptor activity during the perinatal period is important for development of testosterone sensitivity and aggressive behavior in adulthood [111], which could be impacted by reduced expression of 5-HT_1A_ receptors during the neonatal period, as has been observed in PC exposed rats [50].

Testosterone can inhibit the function and expression of 5-HT_1A_ receptors [108,109], and androgen-induced increases in aggression are inhibited by 5-HT_1A _receptor agonists [248]. Expression of 5-HT_1A_ receptors is reduced by PC exposure, which could exacerbate the effects of testosterone and lead to a further reduction in receptor function in PC exposed males during and after puberty, when testosterone levels increase to adult levels. Additionally, changes in 5-HT_1A_ receptors in adult males may play an important role in the altered stress response and aggression often observed in males, since such effects may be exacerbated during puberty related to an interaction with 5-HT and testosterone. This idea could be tested by measuring endocrine status in PC exposed males in clinical and preclinical studies. Males also seem to be more sensitive to the interaction between early SERT function and early environmental stress [350,345], suggesting that susceptibility to disruptions in the neonatal period caused by maternal neglect may contribute to the behavioral differences observed in males.

Although the effects of PC exposure appear to be greater in males, for the most part, females have not been studied extensively. Future preclinical studies should include females to determine whether this apparent sex-specific effect is indeed the case. However, differences between males and females in serotonergic signaling must still be carefully considered [108]. 

### 2 Genetic Susceptibility to Prenatal Drug Exposure

An important consideration in the design of future clinical and preclinical studies is the genetic makeup of the subject population. Recent studies have shown that genetic variance in SERT, MAO-A and COMT, impact the effect of prenatal SSRI exposure in infants [76,351]. As previously mentioned, allelic variance exists in several proteins associated with serotonergic signaling, the roles of which have never been investigated in terms of their impact on the effects of PC exposure [41,43]. However, allelic variance in the 5-HT_1A_ receptor has been linked to drug use and withdrawal [352], suggesting that mothers who continue to take cocaine, regardless of negative consequences, may have a genetic susceptibility based on serotonergic signaling that they may be passing on to their children. Such allelic variance has also been associated with aggression [162,353]. SERT polymorphisms with lower functionality, similar to what could occur in PC exposed children, have also been associated with aggression and ADHD [162,353], and males are especially sensitive to early environmental stress [162,354], potentially creating a positive feedback loop leading to poor behavioral outcomes for these children dependent on multiple insults to serotonergic function. Future studies utilizing animal models deficient in serotonergic signaling components would significantly increase understanding of the role genetic variation may play in vulnerability to prenatal drug exposure. 

## CONCLUDING REMARKS

Fig. (**5**) presents a simplified model of how PC exposure induces alterations in embryonic and neonatal serotonergic signaling molecules (including low S100β, SERT and 5-HT_1A_) can result in an overall decrease in 5-HT signaling during infancy. This deficit has long lasting effect on adult 5-HT signaling, as well as HPA activity. Dysregulation of the HPA axis and 5-HT signaling combine to alter stress responsiveness, which interacts with other behavioral problems that stem from serotonergic signaling deficits independent of stress responsivity. These behavioral problems are commonly comorbid and can increase the severity of one another. This could lead to children with multiple behavioral problems that last through adulthood. Fortunately, this model points to several molecular targets for potential pharmacotherapeutic strategies. 

Clearly, the effects of PC exposure on behaviors are complex and are not due solely to changes in serotonergic signaling. The impact of such exposure on the development of the dopaminergic system is dose-, age- and sex- dependent, similar to effects on serotonergic signaling [18,355]. Little is known about how the noradrenergic system is impacted by PC exposure, which should be considered, since cocaine also blocks NET, and it has been suggested that NE may modulate the effects of prenatal SSRI exposure [119]. This is an especially important consideration given the interplay between these monoaminergic signaling systems during brain development. However, it is clear from the available literature that PC exposure has a substantial impact on serotonergic signaling throughout the lifetime of both humans and animal models. Such changes appear to be associated with many of the observed behavioral deficits resulting from prenatal cocaine exposure, which need to be directly tested. Possible avenues for such investigation include:

1. Determining the role of SERT inhibition in complementary clinical and preclinical prenatal SSRI studies;
2. Inclusion of females in all studies of the impact of PC exposure and prenatal SSRI exposure on the developing serotonergic system and related behaviors;
3. In depth investigation of male susceptibility to the detrimental effects of PC exposure and prenatal SSRI exposure, including the role played by the male hormonal milieu;
4. Further investigation of 5-HT_1A_ receptors in increased aggression, depression, attention and social interaction deficits seen in human and animal PC exposed offspring, with an emphasis on potential pharmacologic intervention;
5. Further investigation of the interaction of the early environment created by drug-using parents with drug-exposed infants, which can negatively affect an already vulnerable developing nervous system;
6. Devising and implementing early behavioral interventions with both parents and children that may help reduce the impact of this double insult.

## Figures and Tables

**Fig. (5):**
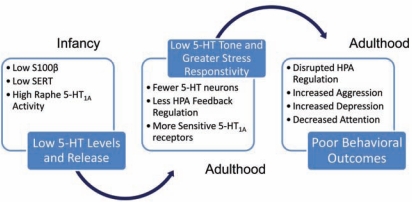
Developmental Effects of Serotonin Deficits. PC exposure can cause a variety of serotonergic changes during infancy that culminate in low serotonergic tone (Box A.) PC exposure causes deficits in adult 5-HT signaling, which may be developmental compensations for early disruptions (Box B). The sum of the 5-HT signaling deficits accrued by adulthood lead to a number of interacting behavioral problems and overall poor behavioral outcomes (Box C).

**Table 1:** Kd Values for Each Drug for Human Serotonin Transporter (SERT), Norepinephrine Transporter (NET) and Dopamine Transporter (DAT)

Drug	K_d_ for SERT	K_d_ for NET	K_d_ for DAT
Cocaine	340	1420	220
Amnefolic Acid	>10,000	>10,000	18.7*
Amitriptyline	4.30	35	3250
Bupropion	9100	52000	520
Citalopram	1.16	4070	28100
Fluoxetine	0.81	240	3600
Fluovoxamine	2.2	1300	9200
Imipramine	1.4	37	8500
Iprindole	1620	1262	6530
Mianserin	4000	71	1000
Nomifensine	1010	15.6	56
Norfluoxetine	1.47	1426	420
Paroxetine	0.13	40	490
Sertraline	0.29	420	25
Venlafaxine	8.9	1060	9300

Adapted from [1] and [2].

* Ki data for rat transporters.

**Table 2:** PC exposure and 5-HT Signaling in Rodent Models

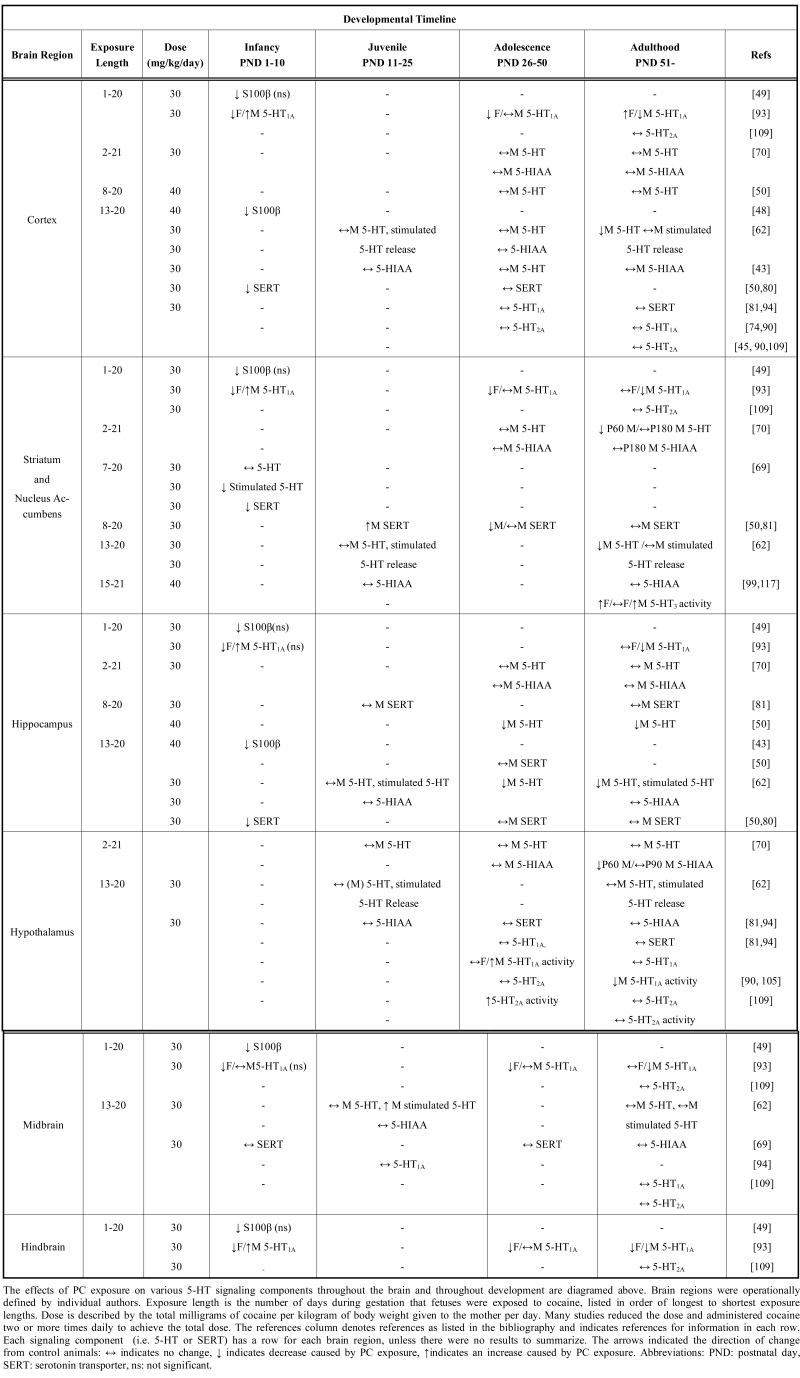

## ABBREVIATIONS

PC: = Prenatal cocaine
GD: = Gestational day
PND: = Postnatal day
5-HT: = 5-hydroxytrytophan (serotonin)
SERT: = Serotonin transporter
SSRI: = Selective serotonin reuptake inhibitor
CNS: = Central nervous system
HPLC: = High-pressure liquid chromatography
PCA: = *p*-chloroamphetamine
CORT: = Cortisol or corticosterone
ACTH: = Adrenocorticotropic hormone
MAO-A: = Monoamine oxidase A
5-HIAA: = 5-Hydroxyindoleacetic acid
CSF: = Cerebrospinal fluid
COMT: = c-O-methyltransferase
ADHD: = Attention-deficit hyperactivity disorder
USV: = Ultrasonic vocalization
AFA: = Amfonelic acid
NE(T): = Norepinephrine (transporter)
DA(T): = Dopamine (transporter)
HPA: = Hypothalamic-pituitary-adrenal
CRF: = Corticotrophin releasing factor
GR: = Glucocorticoid receptors
NGF-I: = Nerve Growth Factor-I
CREB: = CRE Response binding

## References

[1] Mahalik M P, Gautieri R F, Mann D E  (1980). Teratogenic potential of cocaine hydrochloride in CF-1 mice. J. Pharm. Sci.

[2] Chasnoff I J, Burns W J, Schnoll S H, Burns K A (1985). Cocaine use in pregnancy. N. Engl. J. Med.

[3] Chasnoff I J, Bussey M E, Savich R, Stack C M (1986). Perinatal cerebral infarction and maternal cocaine use. J. Pediatr.

[4] Bingol N, Fuchs M, Diaz V, Stone R K, Gromisch D S (1987). Teratogenicity of cocaine in humans. J. Pediatr.

[5] Blakeslee S (1989). Crack's Toll Among Babies: A Joyless View, Even of Toys. The New York Times.

[6] Duke L (1988). Drugs get choke hold in early stages of life: district sees increase in pregnant women who use cocaine, PCP. The Wasington Post.

[7] Kain Z N, Kain T S, Scarpelli E M (1992). Cocaine exposure in utero: perinatal development and neonatal manifestations--review. J. Toxicol. Clin. Toxicol.

[8] Hannig V L, Phillips J (1991). Maternal cocaine abuse and fetal anomalies: evidence for teratogenic effects of cocaine. South Med. J.

[9] Lester B M, LaGasse L L, Seifer R (1998). Cocaine exposure and children: the meaning of subtle effects. Science.

[10] Lester B M, Padbury J F (2009). Third pathophysiology of prenatal cocaine exposure. Dev. Neurosci.

[11] Mathias R (1995). NIDA survey provides first national data on drug use during pregnancy. NIDA Notes.

[12] (2007). Office of Applied Studies. Results from the 2006 National Survey
on Drug Use and Health: National findings (DHHS Publication No.
SMA 07-4293, NSDUH Series H-32).

[13] Behnke M, Eyler F D, Conlon M, Casanova O Q, Woods N S (1997). How fetal cocaine exposure increases neonatal hospital costs. Pediatrics.

[14] Meyer K D, Zhang L (2009). Short- and long-term adverse effects of cocaine abuse during pregnancy on the heart development. Ther. Adv. Cardiovasc. Dis.

[15] Ho J, Afshani E, Stapleton F B (1994). Renal vascular abnormalities associated with prenatal cocaine exposure. Clin. Pediatr. (Phila).

[16] ga C A, Brust J C, Bateman D, Hauser W A (1999). Dose-response effect of fetal cocaine exposure on newborn neurologic function. Pediatrics.

[17] Olsen G D, Murphey L J (1995). Effects of morphine and cocaine on breathing control in neonatal animals: a minireview. NIDA Res. Monogr.

[18] Glatt S J, Bolanos C A, Trksak G H, Jackson D (2000). Effects of prenatal cocaine exposure on dopamine system development: a meta-analysis. Neurotoxicol. Teratol.

[19] Stanwood G D, Washington R A, Shumsky J S, Levitt P (2001). Prenatal cocaine exposure produces consistent developmental alterations in dopamine-rich regions of the cerebral cortex. Neuroscience.

[20] Bakshi K, Gennaro S, Chan C Y, Kosciuk M, Liu J, Stucky A, Trenkner E, Friedman E, Nagele R G, Wang H Y (2009). Prenatal cocaine reduces AMPA receptor synaptic expression through hyperphosphorylation of the synaptic anchoring protein GRIP. J. Neurosci.

[21] McMurray M S, Joyner P W, Middleton C W, Jarrett T M, Elliott D L, Black M A, Hofler V E, Walker C H, Johns J M (2008). Intergenerational effects of cocaine on maternal aggressive behavior and brain oxytocin in rat dams. Stress.

[22] Cipriani A, Brambilla P, Furukawa T, Geddes J, Gregis M, Hotopf M, Malvini L, Barbui C (2005). Fluoxetine versus other types of pharmacotherapy for depression. Cochrane. Database Syst. Rev.

[23] Tatsumi M, Groshan K, Blakely R D, Richelson E (1997). Pharmacological profile of antidepressants and related compounds at human monoamine transporters. Eur. J. Pharmacol.

[24] Cabrera T M, Levy A D, Li Q, Van de Kar L D, Battagha G (1994). Cocaine-induced deficits in ACTH and corticosterone responses in female rat progeny. Brain Res. Bull.

[25] Lisboa S F, Oliveira P E, Costa L C, Venancio E J, Moreira E G (2007). Behavioral evaluation of male and female mice pups exposed to fluoxetine during pregnancy and lactation. Pharmacology.

[26] Oberlander T F, Reebye P, Misri S, Papsdorf M, Kim J, Grunau R E (2007). Externalizing and attentional behaviors in children of depressed mothers treated with a selective serotonin reuptake inhibitor antidepressant during pregnancy. Arch. Pediatr. Adolesc. Med.

[27] Casper R C, Fleisher B E, Lee-Ancajas J C, Gilles A, Gaylor E, DeBattista A, Hoyme H E (2003). Follow-up of children of depressed mothers exposed or not exposed to antidepressant drugs during pregnancy. J. Pediatr.

[28] Bairy K L, Madhyastha S, Ashok K P, Bairy I, Malini S (2007). Developmental and behavioral consequences of prenatal fluoxetine. Pharmacology.

[29] Tuccori M, Testi A, Antonioli L, Fornai M, Montagnani S, Ghisu N, Colucci R, Corona T, Blandizzi C, Del Tacca M (2009). Safety concerns associated with the use of serotonin reuptake inhibitors and other serotonergic/noradrenergic antidepressants during pregnancy: a review. Clin. Ther.

[30] Gentile S, Bellantuono C (2009). Selective serotonin reuptake inhibitor exposure during early pregnancy and the risk of fetal major malformations: focus on paroxetine. J. Clin. Psychiatry.

[31] Alwan S, Friedman J.M (2009). Safety of selective serotonin reuptake inhibitors in pregnancy. CNS Drugs.

[32] Oberlander T F, Gingrich J A, Ansorge M S (2009). Sustained neurobehavioral effects of exposure to SSRI antidepressants during development: molecular to clinical evidence. Clin. Pharmacol. Ther.

[33] Zeskind P S, Oberlander T, Grunau R, Fitzgerald C, Garber K (2005). Continuing effects of prenatal SSRI exposure detected by spectral analysis of infant cry sounds at two months of age. Society for pediatric research.

[34] Barnes N M, Sharp T (1999). A review of central 5-HT receptors and their function. Neuropharmacology.

[35] Whitaker-Azmitia P M, Druse M, Walker P, Lauder J M (1996). Serotonin as a developmental signal. Behav. Brain Res.

[36] Li Q, Muma N A, Van De Kar L D (1996). Chronic fluoxetine induces a gradual desensitization of 5-HT1A receptors: reductions in hypothalamic and midbrain Gi and G(o) proteins and in neuroendocrine responses to a 5-HT1A agonist. J. Pharmacol. Exp. Ther.

[37] Li Q, Muma N A, Battaglia G, Van De Kar L D (1997). Fluoxetine gradually increases [125I]DOI-labelled 5-HT2A/2C receptors in the hypothalamus without changing the levels of Gq- and G11-proteins. Brain Res.

[38] Li Q, Wichems C, Heils A, Lesch K P, Murphy D L (2000). Reduction in the density and expression, but not G-protein coupling, of serotonin receptors (5-HT1A) in 5-HT transporter knock-out mice: gender and brain region differences. J. Neurosci.

[39] Ritz M C, Cone E J, Kuhar M J (1990). Cocaine inhibition of ligand binding at dopamine, norepinephrine and serotonin transporters: a structure-activity study. Life Sci.

[40] Meyer J S, Shearman L P, Collins L M (1996). Monoamine transporters and the neurobehavioral teratology of cocaine. Pharmacol. Biochem. Behav.

[41] Drago A, Ronchi D D, Serretti A (2008). 5-HT1A gene variants and psychiatric disorders: a review of current literature and selection of SNPs for future studies. Int. J. Neuropsychopharmacol.

[42] Chambers J J, Kurrasch-Orbaugh D M, Parker M A, Nichols D E (2001). Enantiospecific synthesis and pharmacological evaluation of a series of super-potent, conformationally restricted 5-HT(2A/2C) receptor agonists. J. Med. Chem.

[43] Tsai S J, Wang Y C, Chen J Y, Hong C J (2003). Allelic variants of the tryptophan hydroxylase (A218C) and serotonin 1B receptor (A-161T) and personality traits. Neuropsychobiology.

[44] Hoyer D, Clarke D E, Fozard J R, Hartig P R, Martin G R, Mylecharane E J, Saxena P R, Humphrey P P (1994). International Union of Pharmacology classification of receptors for 5-hydroxytryptamine (Serotonin). Pharmacol. Rev.

[45] Spear L P, Frambes N A, Kirstein C L (1989). Fetal and maternal brain and plasma levels of cocaine and benzoylecgonine following chronic subcutaneous administration of cocaine during gestation in rats. Psychopharmacology (Berl).

[46] Whitaker-Azmitia P M, Azmitia E C (1994). Astroglial 5-HT1a receptors and S-100 beta in development and plasticity. Perspect. Dev. Neurobiol.

[47] Lidov H G, Molliver M E (1982). Immunohistochemical study of the development of serotonergic neurons in the rat CNS. Brain Res. Bull.

[48] Wallace J A, Lauder J M (1983). Development of the serotonergic system in the rat embryo: an immunocytochemical study. Brain Res. Bull.

[49] Lauder J M, Bloom F E (1974). Ontogeny of monoamine neurons in
the locus coeruleus, Raphe nuclei and substantia nigra of the rat. I.
Cell differentiation. J. Comp. Neurol.

[50] Johns J M, Lubin D A, Lieberman J A, Lauder J M (2002). Developmental effects of prenatal cocaine exposure on 5-HT1A receptors in male and female rat offspring. Dev. Neurosci.

[51] Kessler F H, Woody G, Portela L V, Tort A B, De Boni R, Peuker A C, Genro V, von Diemen L, de Souza D O, Pechansky F (2007). Brain injury markers (S100B and NSE) in chronic cocaine dependents. Rev. Bras. Psiquiatr.

[52] Akbari H M, Whitaker-Azmitia P M, Azmitia E C (1994). Prenatal cocaine decreases the trophic factor S-100 beta and induced microcephaly: reversal by postnatal 5-HT1A receptor agonist. Neurosci. Lett.

[53] Johns J M, Noonan L R, Zimmerman L I, McMillen B A, Means L W, Walker C H, Lubin D A, Meter K E, Nelson C J, Pedersen C A, Mason G A, Lauder J M (1998). Chronic cocaine treatment alters social/aggressive behavior in Sprague-Dawley rat dams and in their prenatally exposed offspring. Ann. N. Y. Acad. Sci.

[54] Akbari H M, Kramer H K, Whitaker-Azmitia P M, Spear L P, Azmitia E C (1992). Prenatal cocaine exposure disrupts the development of the serotonergic system. Brain Res.

[55] Clarke C, Clarke K, Muneyyirci J, Azmitia E, Whitaker-Azmitia P M (1996). Prenatal cocaine delays astroglial maturation: immunodensitometry shows increased markers of immaturity (vimentin and GAP-43) and decreased proliferation and production of the growth factor S-100. Brain Res. Dev. Brain Res.

[56] Pawluski J L, Galea L A, Brain U, Papsdorf M, Oberlander T F (2009). Neonatal S100B protein levels after prenatal exposure to selective serotonin reuptake inhibitors. Pediatrics.

[57] Tramontina A C, Tramontina F, Bobermin L D, Zanotto C, Souza D F, Leite M C, Nardin P, Gottfried C, Goncalves C A (2008). Secretion of S100B, an astrocyte-derived neurotrophic protein, is stimulated by fluoxetine via a mechanism independent of serotonin. Prog. Neuropsychopharmacol. Biol. Psychiatry.

[58] Jang B S, Kim H, Lim S W, Jang K W, Kim D K (2008). Serum S100B Levels and major depressive disorder: Its Characteristics and role in antidepressant response. Psychiatry Investig.

[59] Schroeter M L, Abdul-Khaliq H, Diefenbacher A, Blasig I E (2002). S100B is increased in mood disorders and may be reduced by antidepressive treatment. Neuroreport.

[60] Nassogne M C, Gressens P, Evrard P, Courtoy P J (1998). In contrast to cocaine, prenatal exposure to methadone does not produce detectable alterations in the developing mouse brain. Brain Res. Dev. Brain Res.

[61] Donato R, Sorci G, Riuzzi F, Arcuri C, Bianchi R, Brozzi F, Tubaro C, Giambanco I (2009). S100B's double life: intracellular regulator and extracellular signal. Biochim. Biophys. Acta.

[62] Rothermundt M, Ponath G, Arolt V (2004). S100B in schizophrenic psychosis. Int. Rev. Neurobiol.

[63] Whitaker-Azmitia P M (2001). Serotonin and brain development: role in human developmental diseases. Brain Res. Bull.

[64] Yang K, Xie G R, Hu Y Q, Mao F Q, Su L Y (2008). The effects of gender and numbers of depressive episodes on serum S100B levels in patients with major depression. J. Neural Transm.

[65] Machado-Vieira R, Lara D R, Portela L V, Goncalves C A, Soares J C, Kapczinski F, Souza D O (2002). Elevated serum S100B protein in drug-free bipolar patients during first manic epi-sode: a pilot study. Eur. Neuropsychopharmacol.

[66] Cabrera-Vera T M, Garcia F, Pinto W, Battaglia G (2000). Neurochemical changes in brain serotonin neurons in immature and adult offspring prenatally exposed to cocaine. Brain Res.

[67] Song J, Guan X W, Ren J Q, He W (2002). Developmental toxicity of cocaine exposure in mid-pregnancy mice. Acta Pharmacol. Sin.

[68] Rudnick G, Wall S C (1992). p-Chloroamphetamine induces serotonin release through serotonin transporters. Biochemistry.

[69] ch H (1991). The serotonin syndrome. Am. J. Psychiatry.

[70] Bruning G, Liangos O, Baumgarten H G (1997). Prenatal development of the serotonin transporter in mouse brain. Cell Tissue Res.

[71] Shuey D L, Sadler T W, Lauder J M (1992). Serotonin as a regulator of craniofacial morphogenesis: site specific malformations following exposure to serotonin uptake inhibitors. Teratology.

[72] Shuey D L, Sadler T W, Tamir H, Lauder J M (1993). Serotonin and morphogenesis. Transient expression of serotonin uptake and binding protein during craniofacial morphogenesis in the mouse. Anat. Embryol. (Berl).

[73] Yavarone M S, Shuey D L, Sadler T W, Lauder J M (1993). Serotonin uptake in the ectoplacental cone and placenta of the mouse. Placenta.

[74] Yan Q (2002). Reduced serotonin release and serotonin uptake sites in the rat nucleus accumbens and striatum after prenatal cocaine exposure. Brain Res.

[75] Laine K, Heikkinen T, Ekblad U, Kero P (2003). Effects of exposure to selective serotonin reuptake inhibitors during pregnancy on serotonergic symptoms in newborns and cord blood monoamine and prolactin concentrations. Arch. Gen. Psychiatry.

[76] Hilli J, Heikkinen T, Rontu R, Lehtimaki T, Kishida I, Aklillu E, Bertilsson L, Vahlberg T, Laine K (2009). MAO-A and COMT genotypes as possible regulators of perinatal serotonergic symptoms after in utero exposure to SSRIs. Eur. Neuropsychopharmacol.

[77] Henderson M G, McMillen B A (1993). Changes in dopamine, serotonin and their metabolites in discrete brain areas of rat offspring after in utero exposure to cocaine or related drugs. Teratology.

[78] Cabrera-Vera T M, Garcia F, Pinto W, Battaglia G (1997). Effect of prenatal fluoxetine (Prozac) exposure on brain serotonin neurons in prepubescent and adult male rat offspring. J. Pharmacol. Exp. Ther.

[79] Tuinier S, Verhoeven W M, van Praag H M (1995). Cerebrospinal fluid 5-hydroxyindolacetic acid and aggression: a critical reappraisal of the clinical data. Int. Clin. Psychopharmacol.

[80] Asberg M (1997). Neurotransmitters and suicidal behavior. The evidence from cerebrospinal fluid studies. Ann. N. Y. Acad. Sci.

[81] Needlman R, Zuckerman B, Anderson G M, Mirochnick M, Cohen D J (1993). Cerebrospinal fluid monoamine precursors and metabolites in human neonates following in utero cocaine exposure: a preliminary study. Pediatrics.

[82] Macedo T, Ribeiro C A, Cotrim D, Tavares P, Morgadinho M T, Caramona M, Vicente M T, Rodrigues L, Cardoso M G, Keating M L (1995). Catecholamine and MHPG plasma levels, platelet MAO activity, and 3H-imipramine binding in heroin and cocaine addicts. Mol. Neurobiol.

[83] Nicotra A, Pierucci F, Parvez H, Senatori O (2004). Monoamine oxidase expression during development and aging. Neurotoxicology.

[84] Cipriani A, Geddes J R, Furukawa T A, Barbui C (2007). Metareview on short-term effectiveness and safety of antidepressants for depression: an evidence-based approach to inform clinical practice. Can. J. Psychiatry.

[85] Battaglia G, Cabrera T M (1994). Potentiation of 5-HT1A receptor-mediated neuroendocrine responses in male but not female rat progeny after prenatal cocaine: evidence for gender differences. J. Pharmacol. Exp. Ther.

[86] McReynolds A M, Meyer J S (1998). Effects of prenatal cocaine exposure on serotonin and norepinephrine transporter density in the rat brain. Ann. N. Y. Acad. Sci.

[87] Montero D, de Ceballos M L, Del Rio J (1990). Down-regulation of 3H-imipramine binding sites in cerebral cortex after prenatal exposure to antidepressants. Life Sci.

[88] Cabrera-Vera T M, Battaglia G (1998). Prenatal exposure to fluoxetine (Prozac) produces site-specific and age-dependent alterations in brain serotonin transporters in rat progeny: evidence from autoradiographic studies. J. Pharmacol. Exp. Ther.

[89] Jovanovic H, Lundberg J, Karlsson P, Cerin A, Saijo T, Varrone A, Halldin C, Nordstrom A L (2008). Sex differences in the serotonin 1A receptor and serotonin transporter binding in the human brain measured by PET. Neuroimage.

[90] Vigod S N, Stewart D E (2009). Emergent research in the cause of mental illness in women across the lifespan. Curr. Opin. Psychiatry.

[91] Forcelli P A, Heinrichs S C (2008). Teratogenic effects of maternal antidepressant exposure on neural substrates of drug-seeking behavior in offspring. Addict. Biol.

[92] Barr C S, Newman T K, Schwandt M, Shannon C, Dvoskin R L, Lindell S G, Taubman J, Thompson B, Champoux M, Lesch K P, Goldman D, Suomi S J, Higley J D (2004). Sexual dichotomy of an interaction between early adversity and the serotonin transporter gene promoter variant in rhesus macaques. Proc. Natl. Acad. Sci. U.S.A.

[93] Sjoberg R L, Nilsson K W, Nordquist N, Ohrvik J, Leppert J, Lindstrom L, Oreland L (2006). Development of depression: sex and the interaction between environment and a promoter polymorphism of the serotonin transporter gene. Int. J. Neuropsychopharmacol.

[94] Miquel M C, Doucet E, Riad M, Adrien J, Verge D, Hamon M (1992). Effect of the selective lesion of serotoninergic neurons on the regional distribution of 5-HT1A receptor mRNA in the rat brain. Brain Res. Mol. Brain Res.

[95] Centenaro L A, Vieira K, Zimmermann N, Miczek K A, Lucion A B, De Almeida R M (2008). Social instigation and aggressive behavior in mice: role of 5-HT1A and 5-HT1B receptors in the prefrontal cortex. Psychopharmacology (Berl).

[96] Fuller R W (1996). Serotonin receptors involved in regulation of pituitary-adrenocortical function in rats. Behav. Brain Res.

[97] Cabrera T M, Yracheta J M, Li Q, Levy A D, Van De Kar L D, Battaglia G (1993). Prenatal cocaine produces deficits in serotonin mediated neuroendocrine responses in adult rat progeny: evidence for long-term functional alterations in brain serotonin pathways. Synapse.

[98] Vicentic A, Cabrera-Vera T M, Pinto W, Battaglia G (2000). 5-HT(1A) and 5-HT(2A) serotonin receptor turnover in adult rat offspring prenatally exposed to cocaine. Brain Res.

[99] Li Q, Muma N A, Battaglia G, Van De Kar L D (1997). A desensitization of hypothalamic 5-HT1A receptors by repeated injections of paroxetine: reduction in the levels of G(i) and G(o) proteins and neuroendocrine responses, but not in the density of 5-HT1A receptors. J. Pharmacol. Exp. Ther.

[100] Chen Z, Tetzlaff J, Sripathirathan K, Carrasco G A, Shankaran M, Van De Kar L D, Muma N A, Battaglia G (2005). Paroxetine is effective in desensitizing 5-HT1A receptor function in adult offspring exposed prenatally to cocaine. Psychopharmacology (Berl).

[101] da Silva V A, Altenburg S P, Malheiros L R, Thomaz T G, Lindsey C J (1999). Postnatal development of rats exposed to fluoxetine or venlafaxine during the third week of pregnancy. Braz. J. Med. Biol. Res.

[102] Azmitia E C, Gannon P J, Kheck N M, Whitaker-Azmitia P M (1996). Cellular localization of the 5-HT1A receptor in primate brain neurons and glial cells. Neuropsychopharmacology.

[103] Kia H K, Brisorgueil M J, Hamon M, Calas A, Verge D (1996). Ultrastructural localization of 5-hydroxytryptamine1A receptors in the rat brain. J. Neurosci. Res.

[104] Jorgensen H S (2007). Studies on the neuroendocrine role of serotonin. Dan. Med. Bull.

[105] Calogero A E, Bagdy G, Szemeredi K, Tartaglia M E, Gold P W, Chrousos G P (1990). Mechanisms of serotonin receptor agonist-induced activation of the hypothalamic-pituitary-adrenal axis in the rat. Endocrinology.

[106] Maswood S, Stewart G, Uphouse L (1995). Gender and estrous cycle effects of the 5-HT1A agonist, 8-OH-DPAT, on hypothalamic serotonin. Pharmacol. Biochem. Behav.

[107] Bolanos C A, Trksak G H, Cohen O S, Jackson D (2002). Differential serotonergic inhibition of *in vitro* striatal [3H] acetylcholine release in prenatally cocaine-exposed male and female rats. Prog. Neuropsychopharmacol. Biol. Psychiatry.

[108] Zhang L, Ma W, Barker J L, Rubinow D R (1999). Sex differences in expression of serotonin receptors (subtypes 1A and 2A) in rat brain: a possible role of testosterone. Neuroscience.

[109] Gogos A, van den B M (2003). Castration reduces the effect of serotonin-1A receptor stimulation on prepulse inhibition in rats. Behav. Neurosci.

[110] Raum W J, McGivern R F, Peterson M A, Shryne J H, Gorski R A (1990). Prenatal inhibition of hypothalamic sex steroid uptake by cocaine: effects on neurobehavioral sexual differentiation in male rats. Brain Res. Dev. Brain Res.

[111] Albonetti E, Gonzalez M I, Siddiqui A, Wilson C A, Farabollini F (1996). Involvement of the 5-HT1A subtype receptor in the neonatal organization of agonistic behaviour in the rat. Pharmacol. Biochem. Behav.

[112] Battaglia G, Cabrera-Vera T M, Van De Kar L D (2000). Prenatal cocaine exposure potentiates 5-HT(2a) receptor function in male and female rat offspring. Synapse.

[113] Maier W, Mossner R, Quednow B B, Wagner M, Hurlemann R (2008). From genes to psychoses and back: the role of the 5HT2alpha-receptor and prepulse inhibition in schizophrenia. Eur. Arch. Psychiatry Clin. Neurosci.

[114] Dwivedi Y, Mondal A C, Payappagoudar G V, Rizavi H S (2005). Differential regulation of serotonin (5HT)2A receptor mRNA and protein levels after single and repeated stress in rat brain: role in learned helplessness behavior. Neuropharmacology.

[115] Nomura M, Nomura Y (2006). Psychological, neuroimaging, and biochemical studies on functional association between impulsive behavior and the 5-HT2A receptor gene polymorphism in humans. Ann. N. Y. Acad. Sci.

[116] Henderson M G, McConnaughey M M, McMillen B A (1991). Long-term consequences of prenatal exposure to cocaine or related drugs: effects on rat brain monoaminergic receptors. Brain Res. Bull.

[117] Cabrera T M, Battaglia G (1994). Delayed decreases in brain 5-hydroxytryptamine2A/2C receptor density and function in male rat progeny following prenatal fluoxetine. J. Pharmacol. Exp. Ther.

[118] Romero G, Toscano E, Del Rio J (1994). Effect of prenatal exposure to antidepressants on 5-HT-stimulated phosphoinositide hydrolysis and 5-HT2 receptors in rat brain. Gen. Pharmacol.

[119] de Ceballos M L, Benedi A, Urdin C, Del Rio J (1985). Prenatal exposure of rats to antidepressant drugs down-regulates beta-adrenoceptors and 5-HT2 receptors in cerebral cortex. Lack of correlation between 5-HT2 receptors and serotonin-mediated behaviour. Neuropharmacology.

[120] Williams S K, Jarrett T M, Lycan T, McMurray M S, Riday T, Obispo-Peak I, Lauder J M, Johns J M (2006). Effects of prenatal cocaine exposure on 5-HT2A receptors in adult rats. Abstr.- Soc. Neurosci.

[121] Chen Z, Waimey K, Van De Kar L D, Carrasco G A, Landry M, Battaglia G (2004). Prenatal cocaine exposure potentiates paroxetine-induced desensitization of 5-HT2A receptor function in adult male rat offspring. Neuropharmacology.

[122] Poncelet M, Perio A, Simiand J, Gout G, Soubrie P, Le Fur G (1995). Antidepressant-like effects of SR 57227A, a 5-HT3 receptor agonist, in rodents. J. Neural Transm. Gen. Sect.

[123] Engleman E A, Rodd Z A, Bell R L, Murphy J M (2008). The role of 5-HT3 receptors in drug abuse and as a target for pharmacotherapy. CNS Neurol. Disord. Drug Targets.

[124] Cervantes M C, Delville Y (2009). Serotonin 5-HT1A and 5-HT3 receptors in an impulsive-aggressive phenotype. Behav. Neurosci.

[125] Bolanos C A, Trksak G H, Glatt S J, Jackson D (2000). Prenatal cocaine exposure increases serotonergic inhibition of electrically evoked acetylcholine release from rat striatal slices at adulthood. Synapse.

[126] Lesch K P (2001). Serotonergic gene expression and depression: implications for developing novel antidepressants. J. Affect. Disord.

[127] Alenina N, Bashammakh S, Bader M (2006). Specification and differentiation of serotonergic neurons. Stem Cell Rev.

[128] Goridis C, Rohrer H (2002). Specification of catecholaminergic and serotonergic neurons. Nat. Rev. Neurosci.

[129] Koebbe M J, Golden J A, Bennett G, Finnell R H, Mackler S A (1999). Effects of prenatal cocaine exposure on embryonic expression of sonic hedgehog. Teratology.

[130] Fox M A, Andrews A M, Wendland J R, Lesch K P, Holmes A, Murphy D L (2007). A pharmacological analysis of mice with a targeted disruption of the serotonin transporter. Psychopharmacology (Berl).

[131] Akimova E, Lanzenberger R, Kasper S (2009). The serotonin-1A receptor in anxiety disorders. Biol. Psychiatry.

[132] Ase A R, Reader T A, Hen R, Riad M, Descarries L (2001). Regional changes in density of serotonin transporter in the brain of 5-HT1A and 5-HT1B knockout mice, and of serotonin innervation in the 5-HT1B knockout. J. Neurochem.

[133] Chae S M, Covington C Y (2009). Biobehavioral outcomes in adolescents and young adults prenatally exposed to cocaine: evidence from animal models. Biol. Res. Nurs.

[134] Frank D A, Augustyn M, Knight W G, Pell T, Zuckerman B (2001). Growth, development, and behavior in early childhood following prenatal cocaine exposure: a systematic review. JAMA.

[135] Delaney-Black V, Covington C, Templin T, Kershaw T, Nordstrom-Klee B, Ager J, Clark N, Surendran A, Martier S, Sokol R J (2000). Expressive language development of children 
exposed to cocaine prenatally: literature review and report of a 
prospective cohort study. J. Commun. Disord.

[136] Spear L P, Silveri M M, Casale M, Katovic N M, Campbell J O, Douglas L A (2002). Cocaine and development: a retrospective perspective. Neurotoxicol. Teratol.

[137] Whitaker-Azmitia P M (2005). Behavioral and cellular consequences of increasing serotonergic activity during brain development: a role in autism? Int. J. Dev. Neurosci.

[138] Lord C, Cook E H, Leventhal B L, Amaral D G (2000). Autism spectrum disorders. Neuron.

[139] Mayes L C (2002). A behavioral teratogenic model of the impact of prenatal cocaine exposure on arousal regulatory systems. Neurotoxicol. Teratol.

[140] Mayes L C, Grillon C, Granger R, Schottenfeld R (1998). Regulation of arousal and attention in preschool children exposed to cocaine prenatally. Ann. N. Y. Acad. Sci.

[141] Coles C D, Bard K A, Platzman K A, Lynch M E (1999). Attentional response at eight weeks in prenatally drug-exposed and preterm infants. Neurotoxicol. Teratol.

[142] Bendersky M, Lewis M (1998). Arousal modulation in cocaine-exposed infants. Dev. Psychol.

[143] Wilens T E, Spencer T J (2010). Understanding attention-deficit/hyperactivity disorder from childhood to adulthood. Postgrad. Med.

[144] Hughes R N (2004). The value of spontaneous alternation behavior (SAB) as a test of retention in pharmacological investigations of memory. Neurosci. Biobehav. Rev.

[145] Karmel B Z, Gardner J.M (1996). Prenatal cocaine exposure effects on arousal-modulated attention during the neonatal period. Dev. Psychobiol.

[146] Zeskind P S, Schuetze P, Coles C D, Platzman K (1993). Prenatal cocaine exposure and infant cry reactivity: A longitudinal analysis in the newborn period. Society for research in Child 
Development.

[147] Accornero V H, Amado A J, Morrow C E, Xue L, Anthony J C, Bandstra E S (2007). Impact of prenatal cocaine exposure on attention and response inhibition as assessed by continuous performance tests. J. Dev. Behav. Pediatr.

[148] Kable J A, Coles C D, Lynch M E, Platzman K (2008). Physiological responses to social and cognitive challenges in 8-year olds with a history of prenatal cocaine exposure. Dev. Psychobiol.

[149] Linares T J, Singer L T, Kirchner H L, Short E J, Min M O, Hussey P, Minnes S (2006). Mental health outcomes of cocaine-exposed children at 6 years of age. J. Pediatr. Psychol.

[150] Bada H S, Langer J, Twomey J, Bursi C, LaGasse L, Bauer C R, Shankaran S, Lester B M, Higgins R, Maza P L (2008). Importance of stability of early living arrangements on behavior outcomes of children with and without prenatal drug exposure. J. Dev. Behav. Pediatr.

[151] Li Z, Coles C D, Lynch M E, Hamann S, Peltier S, LaConte S, Hu X (2009). Prenatal cocaine exposure alters emotional arousal regulation and its effects on working memory. Neurotoxicol. Teratol.

[152] Li Z, Santhanam P, Coles C D, Lynch M E, Hamann S, Peltier S, Hu X (2010). Increased "default mode" activity in adolescents prenatally exposed to cocaine. Hum. Brain Mapp.

[153] Smith R F, Mattran K M, Kurkjian M F, Kurtz S L (1989). Alterations in offspring behavior induced by chronic prenatal cocaine dosing. Neurotoxicol. Teratol.

[154] Johns J M, Means M J, Anderson D R, Bass E W, Means L W, McMillen B A (1992). Prenatal exposure to cocaine: II. Effects
on open field activity and cognitive behavior in Sprague-
Dawley rats. Neurotoxicol. Teratol.

[155] Zeskind P S, Stephens L E (2004). Maternal selective serotonin reuptake inhibitor use during pregnancy and newborn neurobehavior. Pediatrics.

[156] Figueroa R (2010). Use of antidepressants during pregnancy and risk of attention-deficit/hyperactivity disorder in the offspring. J. Dev. Behav. Pediatr.

[157] Vorhees C V, Acuff-Smith K D, Schilling M A, Fisher J E, Moran M S, Buelke-Sam J (1994). A developmental neurotoxicity evaluation of the effects of prenatal exposure to fluoxetine in rats. Fundam. Appl. Toxicol.

[158] Gendle M H, Strawderman M S, Mactutus C F, Booze R M, Levitsky D A, Strupp B J (2003). Impaired sustained attention and altered reactivity to errors in an animal model of prenatal cocaine exposure. Brain Res. Dev. Brain Res.

[159] Gendle M H, White T L, Strawderman M, Mactutus C F, Booze R M, Levitsky D A, Strupp B J (2004). Enduring effects of prenatal cocaine exposure on selective attention and reactivity to errors: evidence from an animal model. Behav. Neurosci.

[160] Wilkins A S, Jones K, Kosofsky B E (1998). Transplacental cocaine
exposure. 2: Effects of cocaine dose and gestational timing. Neurotoxicol. Teratol.

[161] Brunzell D H, Ayres J J, Meyer J S (2002). Effects of prenatal cocaine exposure on latent inhibition in 1-year-old female rats. Pharmacol. Biochem. Behav.

[162] Retz W, Rosler M (2009). The relation of ADHD and violent aggression: What can we learn from epidemiological and genetic studies? Int. J. Law Psychiatry.

[163] Hofvander B, Ossowski D, Lundstrom S, Anckarsater H (2009). Continuity of aggressive antisocial behavior from childhood to adulthood: The question of phenotype definition. Int. J. Law Psychiatry.

[164] Dalley J W, Mar A C, Economidou D, Robbins T W (2008). Neurobehavioral mechanisms of impulsivity: fronto-striatal systems and functional neurochemistry. Pharmacol. Biochem. Behav.

[165] Winstanley C A, Theobald D E, Dalley J W, Cardinal R N, Robbins T W (2006). Double dissociation between serotonergic and dopaminergic modulation of medial prefrontal and orbitofrontal cortex during a test of impulsive choice. Cereb. Cortex.

[166] Dommett E J, Overton P G, Greenfield S A (2009). Drug therapies for attentional disorders alter the signal-to-noise ratio in the superior colliculus. Neuroscience.

[167] Pompeiano M, Palacios J M, Mengod G (1992). Distribution and cellular localization of mRNA coding for 5-HT1A receptor in the rat brain: correlation with receptor binding. J. Neurosci.

[168] Winstanley C A, Theobald D E, Dalley J W, Robbins T W (2005). Interactions between serotonin and dopamine in the control of impulsive choice in rats: therapeutic implications for impulse control disorders. Neuropsychopharmacology.

[169] Spencer T J, Biederman J, Madras B K, Faraone S V, Dougherty D D, Bonab A A, Fischman A J (2005). In vivo neuroreceptor imaging in attention-deficit/hyperactivity disorder: a focus on the dopamine transporter. Biol. Psychiatry.

[170] Bayer L E, Brown A, Mactutus C F, Booze R M, Strupp B J (2000). Prenatal cocaine exposure increases sensitivity to 
the attentional effects of the dopamine D1 agonist SKF81297. J. Neurosci.

[171] Daviss W B (2008). A review of co-morbid depression in pediatric ADHD: etiology, phenomenology, and treatment. J. Child Adolesc. Psychopharmacol.

[172] Alex K D, Pehek E A (2007). Pharmacologic mechanisms of serotonergic regulation of dopamine neurotransmission. Pharmacol. Ther.

[173] Oberlander T F, Grunau R, Mayes L, Riggs W, Rurak D, Papsdorf M, Misri S, Weinberg J (2008). Hypothalamic-pituitary-adrenal (HPA) axis function in 3-month old infants with prenatal selective serotonin reuptake inhibitor (SSRI) antidepressant exposure. Early Hum. Dev.

[174] Hamilton M (1960). A rating scale for depression. J. Neurol. Neurosurg. Psychiatry.

[175] Pellow S, Chopin P, File S E, Briley M (1985). Validation of open:closed arm entries in an elevated plus-maze as a measure of anxiety in the rat. J. Neurosci. Methods.

[176] Ramos A (2008). Animal models of anxiety: do I need multiple tests?. Trends Pharmacol. Sci.

[177] Pollak D D, Rey C E, Monje F J (2010). Rodent models in depression research: classical strategies and new directions. Ann. Med.

[178] Blumberg M S, Sokoloff G (2001). Do infant rats cry?. Psychol. Rev.

[179] Blumberg M S, Alberts J R (1990). Ultrasonic vocalizations by rat pups in the cold: an acoustic by-product of laryngeal braking?. Behav. Neurosci.

[180] Brunelli S A, Aviles J A, Gannon K S, Branscomb A, Shacham S (2009). PRX-00023, a selective serotonin 1A receptor agonist, reduces ultrasonic vocalizations in infant rats bred for high infantile anxiety. Pharmacol. Biochem. Behav.

[181] Fish E W, Faccidomo S, Gupta S, Miczek K A (2004). Anxiolytic-like effects of escitalopram, citalopram, and R-citalopram in maternally separated mouse pups. J. Pharmacol. Exp. Ther.

[182] Hammerness P, Geller D, Petty C, Lamb A, Bristol E, Biederman J (2010). Does ADHD moderate the manifestation of anxiety disorders in children? Eur. Child Adolesc. Psychiatry.

[183] Cox E T, Jones G, Williams S K, McMurray M S, Jamieson-Drake A. Zeskind, P. S. Hodge C, Grewen K M, Johns J M (2010). Prenatal cocaine exposure alters human and rodent infant vocalizations: implications for maternal care and neural integrity. Proc. Intl. Behav. Neurosci. Soc.

[184] Cox E T, Sheikh M, Williams S K, McMurray M S, Johns J M (2009). Prenatal cocaine exposure alters rodent pup ultrasonic vocalizations that might impact the mother-infant relationship: A preliminary study. Abstr.- Soc.Neurosci.

[185] Overstreet D H, Moy S S, Lubin D A, Gause L R, Lieberman J A, Johns J M (2000). Enduring effects of prenatal cocaine administration on emotional behavior in rats. Physiol. Behav.

[186] Johns J M, Noonan L R (1995). Prenatal cocaine exposure affects social behavior in Sprague-Dawley rats. Neurotoxicol. Teratol.

[187] Johns J M, Means M J, Means L W, McMillen B A (1992). Prenatal exposure to cocaine: I. Effects on gestation, development and activity in Sprague-Dawley rats. Neurotoxicol. Teratol.

[188] Church M W, Holmes P A, Overbeck G W, Tilak J P,  
and Zajac C S (1991). Interactive effects of prenatal alcohol and 
cocaine exposures on postnatal mortality, development and 
behavior in the Long-Evans rat. Neurotoxicol. Teratol.

[189] Misri S, Reebye P, Kendrick K, Carter D, Ryan D, Grunau R E, Oberlander T F (2006). Internalizing behaviors in 4-year-old children exposed in utero to psychotropic medications. Am. J. Psychiatry.

[190] Coleman F H, Christensen H D, Gonzalez C L, Rayburn W F (1999). Behavioral changes in developing mice after prenatal 
exposure to paroxetine (Paxil). Am. J. Obstet. Gynecol.

[191] File S E, Tucker J C (1983). Prenatal treatment with clomipramine has an anxiolytic profile in the adolescent rat. Physiol. Behav.

[192] Sobrian S K, Marr L, Ressman K (2003). Prenatal cocaine and/or nicotine exposure produces depression and anxiety in aging rats. Prog. Neuropsychopharmacol. Biol. Psychiatry.

[193] Salas-Ramirez K Y, Frankfurt M, Alexander A, Luine V N, Friedman E (2010). Prenatal cocaine exposure increases anxiety, 
impairs cognitive function and increases dendritic spine density 
in adult rats: influence of sex. Neuroscience.

[194] Gau S S, Ni H C, Shang C Y, Soong W T, Wu Y Y, Lin L Y, Chiu Y N (2010). Psychiatric comorbidity among children and adolescents with and without persistent attention-deficit hyperactivity disorder. Aust. N. Z. J. Psychiatry.

[195] Savitz J, Lucki I, Drevets W C (2009). 5-HT(1A) receptor function in major depressive disorder. Prog. Neurobiol.

[196] Dawson L A, Watson J M (2009). Vilazodone: a 5-HT1A receptor agonist/serotonin transporter inhibitor for the treatment of affective disorders. CNS Neurosci. Ther.

[197] Krishnan V, Nestler E J (2008). The molecular neurobiology of depression. Nature.

[198] Holsboer F, Ising M (2008). Central CRH system in depression and anxiety--evidence from clinical studies with CRH1 receptor antagonists. Eur. J. Pharmacol.

[199] Su S W, Cherng C F, Lin Y C, Yu L (2007). Prenatal exposure of bupropion may enhance agitation, anxiety responses, and sensitivity to cocaine effects in adult mice. Chin J. Physiol.

[200] Herman J P, Cullinan W E (1997). Neurocircuitry of stress: central control of the hypothalamo-pituitary-adrenocortical axis. Trends Neurosci.

[201] Pittenger C, Duman R S (2008). Stress, depression, and neuroplasticity: a convergence of mechanisms. Neuropsychopharmacology.

[202] Muller M B, Zimmermann S, Sillaber I, Hagemeyer T P, Deussing J M, Timpl P, Kormann M S, Droste S K, Kuhn R, Reul J M, Holsboer F, Wurst W (2003). Limbic corticotropin-releasing hormone receptor 1 mediates anxiety-related behavior and hormonal adaptation to stress. Nat. Neurosci.

[203] Spear L P (1996). Assessment of the effects of developmental toxicants: pharmacological and stress vulnerability of offspring. NIDA Res.Monogr.

[204] Magnano C L, Gardner J M, Karmel B Z (1992). Differences in salivary cortisol levels in cocaine-exposed and noncocaine-exposed NICU infants. Dev. Psychobiol.

[205] Jacobson S W, Bihun J T, Chiodo L M (1999). Effects of prenatal alcohol and cocaine exposure on infant cortisol levels. Dev. Psychopathol.

[206] Lester B M, LaGasse L L, Shankaran S, Bada H S, Bauer C R, Lin R, Das A, Higgins R (2010). Prenatal cocaine exposure related to cortisol stress reactivity in 11-Year-old children. J. Pediatr.

[207] Goodwin G A, Bliven T, Kuhn C, Francis R, Spear L P (1997). Immediate early gene expression to examine neuronal activity  following acute and chronic stressors in rat pups: Examination of neurophysiological alterations underlying behavioral consequences of prenatal cocaine exposure. Physiol. Behav.

[208] Battaglia G C T (1994). Potentiation of 5-HT1A Receptor-Mediated neuroendocrine responses in male but not female rat progeny after prental cocaine: evidence for gender differences. J. Pharmacol. Exp. Ther.

[209] Choi S J, Mazzio E A, Reams R R, Soliman K F (1998). Gestational cocaine exposure alters postnatal pituitary-adrenal axis activity and stress endurance in rats. Ann. N. Y. Acad. Sci.

[210] Planeta C S, Berliner J, Russ A, Kosofsky B E (2001). The effect of prenatal cocaine exposure on the stress response of adult mice. Neurotox. Res.

[211] Huber J, Darling S, Park K, Soliman K F (2001). Altered responsiveness to stress and NMDA following prenatal exposure to cocaine. Physiol. Behav.

[212] Lowry C A, Moore F L (2006). Regulation of behavioral responses by corticotropin-releasing factor. Gen. Comp Endocrinol.

[213] Bluet Pajot M T, Mounier F, di Sciullo A, Schmidt B, Kordon C (1995). Differential sites of action of 8OHDPAT, a 5HT1A agonist, on ACTH and PRL secretion in the rat. Neuroendocrinology.

[214] Lowry C A, Hale M W, Plant A, Windle R J, Shanks N, Wood S A, Ingram C D, Renner K J, Lightman S L, Summers C H (2009). Fluoxetine inhibits corticotropin-releasing factor (CRF)-induced behavioural responses in rats. Stress.

[215] Eiden R D, McAuliffe S, Kachadourian L, Coles C, Colder C, Schuetze P (2009). Effects of prenatal cocaine exposure on infant reactivity and regulation. Neurotoxicol. Teratol.

[216] Chaplin T M, Fahy T, Sinha R, Mayes L C (2009). Emotional arousal in cocaine exposed toddlers: prediction of behavior problems. Neurotoxicol. Teratol.

[217] Molitor A, Mayes L C, Ward A (2003). Emotion regulation behavior during a separation procedure in 18-month-old children of mothers using cocaine and other drugs. Dev. Psychopathol.

[218] Dennis T, Bendersky M, Ramsay D, Lewis M (2006). Reactivity and regulation in children prenatally exposed to cocaine. Dev. Psychol.

[219] Spear L P, Kirstein C L, Bell J, Yoottanasumpun V, Greenbaum R, O'Shea J, Hoffmann H, Spear N E (1989). Effects of prenatal cocaine exposure on behavior during the early postnatal period. Neurotoxicol. Teratol.

[220] Heyser C J, McKinzie D L, Athalie F, Spear N E, Spear L P (1994). Effects of prenatal exposure to cocaine on heart rate and nonassociative learning and retention in infant rats. Teratology.

[221] Nulman I, Rovet J, Stewart D E, Wolpin J, Pace-Asciak P, Shuhaiber S, Koren G (2002). Child development following exposure to tricyclic antidepressants or fluoxetine throughout fetal life: a prospective, controlled study. Am. J. Psychiatry.

[222] Wood R D, Molina V A, Wagner J M, Spear L P (1995). Play behavior and stress responsivity in periadolescent offspring exposed prenatally to cocaine. Pharmacol. Biochem. Behav.

[223] Morrow B A, Elsworth J D, Roth R H (2002). Male rats exposed to cocaine in utero demonstrate elevated expression of Fos in the prefrontal cortex in response to environment. Neuropsychopharmacology.

[224] Bilitzke P J, Church M. W (1992). Prenatal cocaine and alcohol exposures affect rat behavior in a stress test (the Porsolt swim test). Neurotoxicol. Teratol.

[225] Molina V A, Wagner J M, Spear L P (1994). The behavioral response to stress is altered in adult rats exposed prenatally to cocaine. Physiol. Behav.

[226] Campbell J O, Bliven T D, Silveri M M, Snyder K J, Spear L P (2000). Effects of prenatal cocaine on behavioral adaptation to chronic stress in adult rats. Neurotoxicol. Teratol.

[227] Johns J M, Knapp D J, Noonan L R (1994). Prenatal exposure to cocaine treatment alters ultrasonic vocalizations to air puffs in adult female rat Offspring. Abstr. Soc. Neurosci.

[228] Hsiao S Y, Cherng C F, Yang Y K, Yeh T L, Yu L (2005). Prenatal bupropion exposure enhances the cocaine reward and stress susceptibility in adult mice. Chin J. Physiol.

[229] Gingras J L, Feibel J B, Dalley L B, Muelenaer A, Knight C G (1995). Maternal polydrug use including cocaine and postnatal infant sleep architecture: preliminary observations and implications for respiratory control and behavior. Early Hum. Dev.

[230] Regalado M G, Schechtman V L, Del Angel A P, Bean X D (1996). Cardiac and respiratory patterns during sleep in cocaine-exposed neonates. Early Hum. Dev.

[231] Scafidi F A, Field T M, Wheeden A, Schanberg S, Kuhn C, Symanski R, Zimmerman E, Bandstra E S (1996). Cocaine-exposed preterm neonates show behavioral and hormonal differences. Pediatrics.

[232] Delville Y, Newman M L, Wommack J C, Taravosh-Lahn K, Cervantes M C (2006). Devel. Aggression.

[233] Blonigen DM, Krueger RF (2006). Human quantitative genetics of aggression.

[234] Blanchard D C, Fukunaga-Stinson C, Takahashi L K,  
Flannelly K J, Blanchard R J (1984). Dominance and aggression in social groups of male and female rats. Behav. Processes.

[235] Bendersky M, Bennett D, Lewis M (2006). Aggression at age 5 as a function of prenatal exposure to cocaine, gender, and environmental risk. J. Pediatr. Psychol.

[236] Bennett D, Bendersky M, Lewis M (2007). Preadolescent health risk behavior as a function of prenatal cocaine exposure and gender. J. Dev. Behav. Pediatr.

[237] Reef J, Diamantopoulou S, van M, I Verhulst F, van der E J (2010). Predicting adult emotional and behavioral problems from externalizing problem trajectories in a 24-year longitudinal study. Eur. Child Adolesc. Psychiatry.

[238] Wood R D, Spear L P (1998). Prenatal cocaine alters social competition of infant, adolescent, and adult rats. Behav. Neurosci.

[239] Estelles J, Rodriguez-Arias M, Maldonado C, Manzanedo C, Aguilar M A, Minarro J (2006). Prenatal cocaine alters later responses to morphine in adult male mice. Prog. Neuropsychopharmacol. Biol. Psychiatry.

[240] Johns J M, Means M J, Bass E W, Means L W, Zimmerman L I, McMillen B A (1994). Prenatal exposure to cocaine: effects on aggression in Sprague-Dawley rats. Dev. Psychobiol.

[241] Singh Y, Jaiswal A K, Singh M, Bhattacharya S K (1998). Effect of prenatal diazepam, phenobarbital, haloperidol and fluoxetine exposure on foot shock induced aggression in rats. Indian J. Exp. Biol.

[242] Miczek K A, Mos J, Olivier B (1989). Brain 5-HT and inhibition of aggressive behavior in animals: 5-HIAA and receptor subtypes. Psychopharmacol. Bull.

[243] Miczek K A, De Almeida R M, Kravitz E A, Rissman E F, de Boer S F, Raine A (2007). Neurobiology of escalated aggression and violence. J. Neurosci.

[244] Carrillo M, Ricci L A, Coppersmith G A, Melloni R H (2009). The effect of increased serotonergic neurotransmission on aggression: a critical meta-analytical review of preclinical studies. Psychopharmacology (Berl).

[245] Ricci L A, Knyshevski I, Melloni R H (2005). Serotonin type 3 receptors stimulate offensive aggression in Syrian hamsters. Behav. Brain Res.

[246] Witte A V, Floel A, Stein P, Savli M, Mien L K, Wadsak W, Spindelegger C, Moser U, Fink M, Hahn A, Mitterhauser M, Kletter K, Kasper S, Lanzenberger R (2009). Aggression is related to frontal serotonin-1A receptor distribution as revealed by PET in healthy subjects. Hum. Brain Mapp.

[247] de Boer S F, Koolhaas J M (2005). 5-HT1A and 5-HT1B receptor agonists and aggression: a pharmacological challenge of the serotonin deficiency hypothesis. Eur. J. Pharmacol.

[248] Ricci L A, Rasakham K, Grimes J M, Melloni R H (2006). Serotonin-1A receptor activity and expression modulate adolescent anabolic/androgenic steroid-induced aggression in hamsters. Pharmacol. Biochem. Behav.

[249] Hutson P H, Sarna G S, O'Connell M T, Curzon G (1989). Hippocampal 5-HT synthesis and release *in vivo* is decreased by infusion of 8-OHDPAT into the nucleus raphe dorsalis. Neurosci. Lett.

[250] Muller C P, Carey R J, Huston J P, Souza Silva M A (2007). Serotonin and psychostimulant addiction: focus on 5-HT1A-receptors. Prog. Neurobiol.

[251] Carey R J, DePalma G, Shanahan A, Damianopoulos E N, Muller C P, Huston J P (2008). Effects on spontaneous and 
cocaine-induced behavior of pharmacological inhibition of 
noradrenergic and serotonergic systems. Pharmacol. Biochem. 
Behav.

[252] Young K A, Liu Y, Wang Z (2008). The neurobiology of social attachment: A comparative approach to behavioral, neuroanatomical, and neurochemical studies. Comp. Biochem. Physiol C. Toxicol. Pharmacol.

[253] Willey A R, Varlinskaya E I, Spear L P (2009). Social interactions and 50 kHz ultrasonic vocalizations in adolescent and adult rats. Behav. Brain Res.

[254] Panksepp J (1981). The ontogeny of play in rats. Dev. Psychobiol.

[255] Pellis S M, Pellis VC (1998). Play fighting of rats in comparative perspective: a schema for neurobehavioral analyses. Neurosci. Biobehav. Rev.

[256] Varlinskaya E I, Spear L P (2004). Acute ethanol withdrawal (hangover) and social behavior in adolescent and adult male and female Sprague-Dawley rats. Alcohol Clin. Exp. Res.

[257] Lewis M W, Phillips G, Bowser M, DeLuca S, Johnson H L, Rosen T S (2009). Cocaine-exposed infant behavior during Still-Face: risk factor analyses. Am. J. Orthopsychiatry.

[258] Davis E, Fennoy I, Laraque D, Kanem N, Brown G, Mitchell J (1992). Autism and developmental abnormalities in children with perinatal cocaine exposure. J. Natl. Med. Assoc.

[259] Jones N A, Field T, Davalos M, Hart S (2004). Greater right frontal EEG asymmetry and nonemphathic behavior are observed in children prenatally exposed to cocaine. Int. J. Neurosci.

[260] Johns J M, Elliott D L, Hofler V E, Joyner P W, McMurray M S, Jarrett T M, Haslup A M, Middleton C L, Elliott J C, Walker C H (2005). Cocaine treatment and prenatal environment interact to disrupt intergenerational maternal behavior in rats. Behav. Neurosci.

[261] Spear L, Snyder K, Krantova Y, Campbell J (2003). Effects of prenatal cocaine exposure and maternal separation on heart rate, orienting response habituation, and retention. Dev. Psychobiol.

[262] Wood R D, Bannoura M D, Johanson I B (1994). Prenatal cocaine exposure: effects on play behavior in the juvenile rat. Neurotoxicol. Teratol.

[263] Johns J M, McMurray M S, Hofler V E, Jarrett T M, Middleton C L, Elliott D L, Mirza R, Haslup A, Elliott J C, Walker C H (2007). Cocaine disrupts pup-induced maternal behavior in juvenile and adult rats. Neurotoxicol. Teratol.

[264] Reiersen A M, Todd R D (2008). Co-occurrence of ADHD and autism spectrum disorders: phenomenology and treatment. Expert Rev. Neurother.

[265] Brodkin E S (2007). BALB/c mice: low sociability and other phenotypes that may be relevant to autism. Behav. Brain Res.

[266] Higley J D, King S T, Hasert M F, Champoux M, Suomi S J, Linnoila M (1996). Stability of interindividual differences in serotonin function and its relationship to severe aggression and competent social behavior in rhesus macaque females. Neuropsychopharmacology.

[267] Maestripieri D, Lindell S G, Ayala A, Gold P W, Higley J D (2005). Neurobiological characteristics of rhesus macaque abusive mothers and their relation to social and maternal behavior. Neurosci. Biobehav. Rev.

[268] Claussen A H, Mundy P C, Mallik S A, Willoughby J C (2002). Joint attention and disorganized attachment status in infants at risk. Dev. Psychopathol.

[269] Sharp H M, Hillenbrand K (2008). Speech and language development and disorders in children. Pediatr. Clin. North Am.

[270] Kuhl P K (2010). Brain mechanisms in early language acquisition. Neuron.

[271] Cone-Wesson B (2005). Prenatal alcohol and cocaine exposure: influ-ences on cognition, speech, language, and hearing. J. Commun. Disord.

[272] Morrow C E, Vogel A L, Anthony J C, Ofir A Y, Dausa A T, Bandstra E S (2004). Expressive and receptive language functioning in preschool children with prenatal cocaine exposure. J. Pediatr. Psychol.

[273] Beeghly M, Martin B, Rose-Jacobs R, Cabral H, Heeren T, Augustyn M, Bellinger D, Frank D A (2006). Prenatal cocaine exposure and children's language functioning at 6 and 9.5 years: moderating effects of child age, birthweight, and gender. J. Pediatr. Psychol.

[274] Lewis B A, Kirchner H L, Short E J, Minnes S, Weishampel P, Satayathum S, Singer L T (2007). Prenatal cocaine and tobacco effects on children's language trajectories. Pediatrics.

[275] Pulsifer M B, Butz A M, O'Reilly F M, Belcher H M (2008). Prenatal drug exposure: effects on cognitive functioning at 5 years of age. Clin. Pediatr.(Phila).

[276] Hurt H, Betancourt L M, Malmud E K, Shera D M, Giannetta J M, Brodsky N L, Farah M J (2009). Children with and without gestational cocaine exposure: a neurocognitive systems analysis. Neurotoxicol. Teratol.

[277] Singer L T, Minnes S, Short E, Arendt R, Farkas K, Lewis B, Klein N, Russ S, Min M O, Kirchner H L (2004). Cognitive outcomes of preschool children with prenatal cocaine exposure. JAMA.

[278] Burke A E, Crenshaw D A, Green J, Schlosser M A, Strocchia-Rivera L (1989). Influence of verbal ability on the expression of aggression in physically abused children. J. Am. Acad. Child Adolesc. Psychiatry.

[279] Brudzynski S M (2005). Principles of rat communication: quantitative parameters of ultrasonic calls in rats. Behav. Genet.

[280] Brunelli S A, Shair H N, Hofer M A (1994). Hypothermic vocalizations of rat pups (Rattus norvegicus) elicit and direct maternal search behavior. J. Comp. Psychol.

[281] Barron S, Hansen-Trench L S, Kaiser D H (1996). Neonatal cocaine exposure and activity rhythms in rats. Behav. Brain Res.

[282] Barron S, Segar T M, Yahr J S, Baseheart B J, Willford J A (2000). The effects of neonatal ethanol and/or cocaine exposure on isolation-induced ultrasonic vocalizations. Pharmacol. Biochem. Behav.

[283] Barron S, Gilbertson R (2005). Neonatal ethanol exposure but not neonatal cocaine selectively reduces specific isolation-induced vocalization waveforms in rats. Behav. Genet.

[284] Hahn M E, Benno R H, Schanz N, Phadia E (2000). The effects of prenatal cocaine exposure and genotype on the ultrasonic calls of infant mice. Pharmacol. Biochem. Behav.

[285] Kehoe P, Boylan C B (1992). Cocaine-induced effects on isolation stress in neonatal rats. Behav. Neurosci.

[286] McMurray M S, Zeskind P S, Moy S S, Jarrett T M, Johns J M (2006). Preliminary effects of *in Utero* cocaine exposure on infant rat ultrasonic vocalizations. Proc. Intl. Behav. Neurosci. Soc.

[287] Williams S K, Cox E T, Heaton C.F. Desai NN, Fay E E, McMurray M S, Johns J M (2010). Gestational cocaine affects maternal motivation and pup preference: Possible dependence on pup vocalizations. Abstr.- Soc. Neurosci.

[288] Olivier B, Molewijk H E, van der Heyden J A, van Oorschot R, Ronken E, Mos J, Miczek K A (1998). Ultrasonic vocalizations in rat pups: effects of serotonergic ligands. Neurosci. Biobehav. Rev.

[289] Hodgson R A, Guthrie D H, Varty G B (2008). Duration of ultrasonic vocalizations in the isolated rat pup as a behavioral measure: sensitivity to anxiolytic and antidepressant drugs. Pharmacol. Biochem. Behav.

[290] Bardin L, Gregoire S, Aliaga M, Malfetes N, Vitton O, Ladure P, Newman-Tancredi A, Depoortere R (2010). Comparison
of milnacipran, duloxetine and pregabalin in the formalin pain test
and in a model of stress-induced ultrasonic vocalizations in rats. Neurosci.

[291] Burgdorf J, Panksepp J, Brudzynski S M, Beinfeld M C, Cromwell H C, Kroes R A, Moskal J R (2009). The effects of selective breeding for differential rates of 50-kHz ultrasonic vocalizations on emotional behavior in rats. Dev. Psychobiol.

[292] Wohr M, Houx B, Schwarting R K, Spruijt B (2008). Effects of experience and context on 50-kHz vocalizations in rats. Physiol. Behav.

[293] Mayes L C, Feldman R, Granger R H, Haynes O M, Bornstein M H, Schottenfeld R (1997). The effects of polydrug use with and without cocaine on mother-infant interaction at 3 and 6 months. Infant Behav. Dev.

[294] Light K C, Smith T E, Johns J M, Brownley K A, Hofheimer J A, Amico J A (2000). Oxytocin responsivity in mothers of infants: a preliminary study of relationships with blood pressure during laboratory stress and normal ambulatory activity. Health Psychol.

[295] Neuspiel D R, Hamel S C, Hochberg E, Greene J, Campbell D (1991). Maternal cocaine use and infant behavior. Neurotoxicol. Teratol.

[296] Minnes S, Singer L T, Arendt R, Satayathum S (2005). Effects of prenatal cocaine/polydrug use on maternal-infant feeding interactions during the first year of life. J. Dev. Behav. Pediatr.

[297] Burns K, Chethik L, Burns W J, Clark R (1991). Dyadic disturbances in cocaine-abusing mothers and their infants. J. Clin. Psychol.

[298] Murphy J M, Jellinek M, Quinn D, Smith G, Poitrast F G, Goshko M (1991). Substance abuse and serious child mistreatment: prevalence, risk, and outcome in a court sample. Child Abuse Negl.

[299] Nair P, Black M M, Schuler M, Keane V, Rigney B A, Magder L (1997). Risk factors for disruption in primary caregiving among infants of substance abusing women. Child Abuse Negl.

[300] Leventhal J M, Forsyth B W, Qi K, Johnson L, Schroeder D, Votto N (1997). Maltreatment of children born to women 
who used cocaine during pregnancy: a population-based study. Pediatrics.

[301] Wasserman D R, Leventhal J M (1993). Maltreatment of children born to cocaine-dependent mothers. Am. J. Dis. Child.

[302] Johns J M, Noonan L R, Zimmerman L I, Li L, Pedersen C A (1997). Effects of short- and long- term withdrawal from gestational cocaine treatment on maternal behavior and aggression in Sprague-Dawley rats. Dev. Neurosci.

[303] Johns J M, Noonan L R, Zimmerman L I, Li L, Pedersen C A (1994). Effects of chronic and acute cocaine treatment on the onset of maternal behavior and aggression in Sprague-Dawley rats. Behav. Neurosci.

[304] Kinsley C H, Turco D, Bauer A, Beverly M, Wellman J, Graham A L (1994). Cocaine alters the onset and maintenance of maternal behavior in lactating rats. Pharmacol. Biochem. Behav.

[305] Elliott J C, Lubin D A, Walker C H, Johns J M (2001). Acute cocaine alters oxytocin levels in the medial preoptic area and amygdala in lactating rat dams: implications for cocaine-induced changes in maternal behavior and maternal aggression. Neuropeptides.

[306] Johns J M, Nelson C J, Meter K E, Lubin D A, Couch C D, Ayers A, Walker C H (1998). Dose-dependent effects of multiple acute cocaine injections on maternal behavior and aggression in Sprague-Dawley rats. Dev. Neurosci.

[307] Vernotica E M, Morrell J I (1998). Plasma cocaine levels and locomotor activity after systemic injection in virgin and in lactating maternal female rats. Physiol. Behav.

[308] Zeskind P S, Platzman K, Coles C D, Schuetze P (1996). Cry analysis detects subclinical effects of prenatal alcohol exposure. Infant Behav. Dev.

[309] Brunk M A, Henggeler S W (1984). Child influences on adult controls: An experimental investigation. Dev. Psychol.

[310] Belsky J (1993). Etiology of child maltreatment: a developmental-ecological analysis. Psychol. Bull.

[311] Stern J M, Mackinnon D A (1978). Sensory regulation of maternal behavior in rats: effects of pup age. Dev. Psychobiol.

[312] Stern J M, Azzara A V (2002). Thermal control of mother-young contact revisited: hyperthermic rats nurse normally. Physiol. Behav.

[313] Stern J M (1997). Offspring-induced nurturance: animal-human parallels. Dev. Psychobiol.

[314] Logsdon M C, Wisner K, Hanusa B H (2009). Does maternal role functioning improve with antidepressant treatment in women with postpartum depression?. J. Womens Health (Larchmt.).

[315] Johns J M, Joyner P W, McMurray M S, Elliott D L, Hofler V E, Middleton C L, Knupp K, Greenhill K W, Lomas L M, Walker C H (2005). The effects of dopaminergic/serotonergic reuptake inhibition on maternal behavior, maternal aggression, and oxytocin in the rat. Pharmacol. Biochem. Behav.

[316] Philip N S, Carpenter L L, Tyrka A R, Price L H (2008). Augmentation of antidepressants with atypical antipsychotics: a review of the current literature. J. Psychiatr. Pract.

[317] Li M, Budin R, Fleming A S, Kapur S (2005). Effects of novel antipsychotics, amisulpiride and aripiprazole, on maternal behavior in rats. Psychopharmacology (Berl).

[318] Lavi-Avnon Y, Yadid G, Overstreet D H, Weller A (2005). Abnormal patterns of maternal behavior in a genetic animal model of depression. Physiol. Behav.

[319] Braw Y, Malkesman O, Merenlender A, Dagan M, Bercovich A, Lavi-Avnon Y, Weller A (2009). Divergent maternal behavioral patterns in two genetic animal models of depression. Physiol. Behav.

[320] Smith J W, Seckl J R, Evans A T, Costall B, Smythe J W (2004). Gestational stress induces post-partum depression-like behaviour and alters maternal care in rats. Psychoneuroendocrinology.

[321] Chen Y, Holzman C, Chung H, Senagore P, Talge N M, Siler-Khodr T (2010). Levels of maternal serum corticotropin-releasing hormone (CRH) at midpregnancy in relation to maternal characteristics. Psychoneuroendocrinology.

[322] Poobalan A S, Aucott L S, Ross L, Smith W C, Helms P J, Williams J H (2007). Effects of treating postnatal depression on mother-infant interaction and child development: systematic review. Br. J. Psychiatry.

[323] Shipman K, Taussig H (2009). Mental health treatment of child abuse and neglect: the promise of evidence-based practice. Pediatr. Clin. North Am.

[324] Briere J, Jordan C E (2009). Childhood maltreatment, intervening variables, and adult psychological difficulties in women: an overview. Trauma Violence Abuse.

[325] Fish E W, Shahrokh D, Bagot R, Caldji C, Bredy T, Szyf M, Meaney M J (2004). Epigenetic programming of stress responses through variations in maternal care. Ann. N. Y. Acad. Sci.

[326] Veenema A H (2009). Early life stress, the development of aggression and neuroendocrine and neurobiological correlates: what can we learn from animal models?. Front Neuroendocrinol.

[327] Stanger C, Higgins S T, Bickel W K, Elk R, Grabowski J, Schmitz J, Amass L, Kirby K C, Seracini A M (1999). Behavioral and emotional problems among children of cocaine- and opiate-dependent parents. J. Am. Acad. Child Adolesc. Psychiatry.

[328] Goodwin G A, Heyser C J, Moody C A, Rajachandran L, Molina V A, Arnold H M, McKinzie D L, Spear N E, Spear L P (1992). A fostering study of the effects of prenatal cocaine exposure: II. Offspring behavioral measures. Neurotoxicol. Teratol.

[329] Pedersen C A, Boccia M L (2002). Oxytocin links mothering received, mothering bestowed and adult stress responses. Stress.

[330] Liu D, Diorio J, Tannenbaum B, Caldji C, Francis D, Freedman A, Sharma S, Pearson D, Plotsky P M, Meaney M J (1997). Maternal care, hippocampal glucocorticoid receptors, and hypothalamic-pituitary-adrenal responses to stress. Science.

[331] Meaney M J (2001). Maternal care, gene expression, and the transmission of individual differences in stress reactivity across generations. Annu. Rev. Neurosci.

[332] Francis D D, Champagne F A, Liu D, Meaney M J (1999). Maternal care, gene expression, and the development of individual differences in stress reactivity. Ann. N. Y. Acad. Sci.

[333] Carter D A, Lightman S L (1987). Oxytocin stress responses are dependent upon emotionality. Psychoneuroendocrinology.

[334] Windle R J, Shanks N, Lightman S L, Ingram C D (1997). Central oxytocin administration reduces stress-induced corticosterone release and anxiety behavior in rats. Endocrinology.

[335] Neumann I D, Torner L, Wigger A (2000). Brain oxytocin: differential inhibition of neuroendocrine stress responses and anxiety-related behaviour in virgin, pregnant and lactating rats. Neuroscience.

[336] Spear L P, Scalzo F M (1985). Ontogenetic alterations in the effects of food and/or maternal deprivation on 5-HT, 5-HIAA and 5-HIAA/5-HT ratios. Brain Res.

[337] Zhang T Y, Meaney M J (2010). Epigenetics and the environmental regulation of the genome and its function. Annu. Rev. Psychol.

[338] Weaver I C, Cervoni N, Champagne F A, D'Alessio A C, Sharma S, Seckl J R, Dymov S, Szyf M, Meaney M J (2004). Epigenetic programming by maternal behavior. Nat. Neurosci.

[339] Weaver I C, D'Alessio A C, Brown S E, Hellstrom I C, Dymov S, Sharma S, Szyf M, Meaney M J (2007). The transcription factor nerve growth factor-inducible protein a mediates epigenetic programming: altering epigenetic marks by immediate-early genes. J. Neurosci.

[340] Weaver I C (2007). Epigenetic programming by maternal behavior and pharmacological intervention. Nature versus nurture: let's call the whole thing off. Epigenetics.

[341] Ichise M, Vines D C, Gura T, Anderson G M, Suomi S J, Higley J D, Innis R B (2006). Effects of early life stress on [11C]DASB positron emission tomography imaging of serotonin transporters in adolescent peer- and mother-reared rhesus monkeys. J. Neurosci.

[342] Shannon C, Schwandt M L, Champoux M, Shoaf S E, Suomi S J, Linnoila M, Higley J D (2005). Maternal absence and stability of individual differences in CSF 5-HIAA concentrations in rhesus monkey infants. Am. J. Psychiatry.

[343] Miller J M, Kinnally E L, Ogden R T, Oquendo M A, Mann J J, Parsey R V (2009). Reported childhood abuse is associated with low serotonin transporter binding *in vivo* in major depressive disorder. Synapse.

[344] Matsuzaki H, Izumi T, Matsumoto M, Togashi H, Yamaguchi T, Yoshida T, Watanabe M, Yoshioka M (2009). Early postnatal stress affects 5-HT1A receptor function in the medial prefrontal cortex in adult rats. Eur. J. Pharmacol.

[345] Oreland S, Pickering C, Gokturk C, Oreland L, Arborelius L, Nylander I (2009). Two repeated maternal separation procedures 
differentially affect brain 5-hydroxytryptamine transporter and 
receptors in young and adult male and female rats. Brain Res.

[346] Roy A (2002). Self-rated childhood emotional neglect and CSF monoamine indices in abstinent cocaine-abusing adults: possible implications for suicidal behavior. Psychiatry Res.

[347] Zanettini C, Carola V, Lo I L, Moles A, Gross C, D'Amato F R (2010). Postnatal handling reverses social anxiety in serotonin receptor 1A knockout mice. Genes Brain Behav.

[348] Field T, Diego M, Hernandez-Reif M, Vera Y, Gil K, Schanberg S, Kuhn C, Gonzalez-Garcia A (2004). Prenatal maternal biochemistry predicts neonatal biochemistry. Int. J. Neurosci.

[349] Araya R, Hu X, Heron J, Enoch M A, Evans J, Lewis G, Nutt D, Goldman D (2009). Effects of stressful life events, maternal depression and 5-HTTLPR genotype on emotional symptoms in pre-adolescent children. Am. J. Med. Genet. B Neuropsychiatr. Genet.

[350] Schwandt M L, Lindell S G, Sjoberg R L, Chisholm K L, Higley J D, Suomi S J, Heilig M, Barr C S (2010). Gene-environment interactions and response to social intrusion in male and female rhesus macaques. Biol. Psychiatry.

[351] Oberlander T F, Bonaguro R J, Misri S, Papsdorf M, Ross C J, Simpson E M (2008). Infant serotonin transporter (SLC6A4) promoter genotype is associated with adverse neonatal outcomes after prenatal exposure to serotonin reuptake inhibitor medications. Mol. Psychiatry.

[352] Lee Y S, Choi S W, Han D H, Kim D J, Joe K H (2009). Clinical manifestation of alcohol withdrawal symptoms related to genetic polymorphisms of two serotonin receptors and serotonin transporter. Eur. Addict. Res.

[353] Popova N K (2008). From gene to aggressive behavior: the role of brain serotonin. Neurosci. Behav. Physiol.

[354] Biederman J (2005). Attention-deficit/hyperactivity disorder: a selective overview. Biol. Psychiatry.

[355] Friedman E, Wang H Y (1998). Prenatal cocaine exposure alters signal transduction in the brain D1 dopamine receptor system. Ann. N. Y. Acad. Sci.

